# Engineering xylose metabolism in thraustochytrid T18

**DOI:** 10.1186/s13068-018-1246-1

**Published:** 2018-09-17

**Authors:** Alexandra Merkx-Jacques, Holly Rasmussen, Denise M. Muise, Jeremy J. R. Benjamin, Haila Kottwitz, Kaitlyn Tanner, Michael T. Milway, Laura M. Purdue, Mark A. Scaife, Roberto E. Armenta, David L. Woodhall

**Affiliations:** Mara Renewables Corporation, 101 Research Drive, Dartmouth, NS B2Y 4T6 Canada

**Keywords:** Thraustochytrid, Xylose metabolism, Xylose isomerase, Metabolic engineering, Biomass production, Lipid production for biofuel

## Abstract

**Background:**

Thraustochytrids are heterotrophic, oleaginous, marine protists with a significant potential for biofuel production. High-value co-products can off-set production costs; however, the cost of raw materials, and in particular carbon, is a major challenge to developing an economical viable production process. The use of hemicellulosic carbon derived from agricultural waste, which is rich in xylose and glucose, has been proposed as a sustainable and low-cost approach. Thraustochytrid strain T18 is a commercialized environmental isolate that readily consumes glucose, attaining impressive biomass, and oil production levels. However, neither thraustochytrid growth capabilities in the presence of xylose nor a xylose metabolic pathway has been described. The aims of this study were to identify and characterize the xylose metabolism pathway of T18 and, through genetic engineering, develop a strain capable of growth on hemicellulosic sugars.

**Results:**

Characterization of T18 performance in glucose/xylose media revealed diauxic growth and copious extracellular xylitol production. Furthermore, T18 did not grow in media containing xylose as the only carbon source. We identified, cloned, and functionally characterized a xylose isomerase. Transcriptomics indicated that this xylose isomerase gene is upregulated when xylose is consumed by the cells. Over-expression of the native xylose isomerase in T18, creating strain XI 16, increased xylose consumption from 5.2 to 7.6 g/L and reduced extracellular xylitol from almost 100% to 68%. Xylose utilization efficiency of this strain was further enhanced by over-expressing a heterologous xylulose kinase to reduce extracellular xylitol to 20%. Moreover, the ability to grow in media containing xylose as a sole sugar was dependent on the copy number of both xylose isomerase and xylulose kinase present. In fed-batch fermentations, the best xylose metabolizing isolate, XI-XK 7, used 137 g of xylose versus 39 g by wild type and produced more biomass and fatty acid.

**Conclusions:**

The presence of a typically prokaryotic xylose isomerase and xylitol production through a typically eukaryotic xylose reductase pathway in T18 is the first report of an organism naturally encoding enzymes from two native xylose metabolic pathways. Our newly engineered strains pave the way for the growth of T18 on waste hemicellulosic feedstocks for biofuel production.

**Electronic supplementary material:**

The online version of this article (10.1186/s13068-018-1246-1) contains supplementary material, which is available to authorized users.

## Background

As the finite source of fossil fuels is recognized, as well as the environmental consequences of greenhouse gas production, the development of sustainable biofuels has become an urgent priority [1]. Potential solutions to this global challenge include the use of photosynthetic microorganisms to produce biomass and lipids. These microorganisms fix carbon dioxide, in the presence of light and nutrients, but are limited by productivity and volumetric yield [2]. Heterotrophic microorganisms present a nearer term solution to this biofuel challenge, efficiently converting fixed carbon into biomass and lipids, with productivities and yields that are commercially relevant. Nonetheless, the cost of carbon feedstocks for the growth of these microorganisms for large-scale biofuel production remains a significant limit to commercialization. Utilization of hemicellulose, an abundant polysaccharide within lignocellulose composed of a mixture of sugars including glucose and xylose, has been proposed as a potential solution for reducing costs [3, 4].

Thraustochytrids are heterotrophic marine protists with a significant biotechnological capacity for lipid production [5–8]. Due to the significant amount of docosahexaenoic acid (DHA), eicosapentaenoic acid (EPA), and total lipids synthesized by some strains, thraustochytrids are often used in the commercial production of nutritional supplements [9] and as candidates for biofuel production [10, 11].

**Fig. 1:**
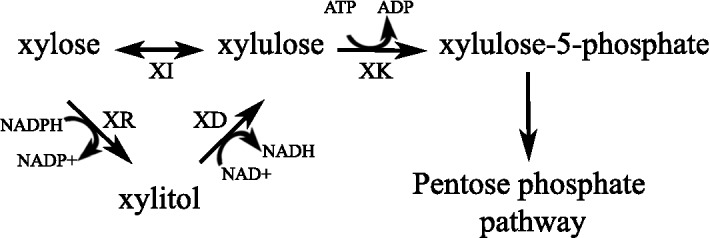
Two common xylose metabolism pathways in microorganisms. Xylose can be directly converted to xylulose by a xylose isomerase (XI) or converted to xylitol by a xylose reductase (XR). Xylitol is then converted to xylulose by a xylitol dehydrogenase (XD). Both the XR and XD reactions require co-factors. Xylulose is then converted to xylulose-5-phosphate by an ATP-dependent xylulose kinase (XK)

Two common xylose metabolism pathways in microorganisms (Fig. 1) include a eukaryote-associated xylose reductase/xylitol dehydrogenase pathway and a prokaryote-associated xylose isomerase pathway [12–15]. A xylulose kinase is required to convert the xylulose produced by both of these pathways to xylulose-5-phosphate which is then shuttled through various metabolic pathways. As thraustochytrids are often isolated from mangroves and produce several extracellular enzymes including polysaccharases, they are thought to play a role in decomposing algal and plant materials, suggesting that they may be capable of growth using a wide range of sugars including xylose [16–18]. Thraustochytrids can grow in the presence of a variety of carbon sources such as glucose, glycerol, fructose, sucrose, maltose, and even ethanol [10, 19–22]; however, little research has been published on how or if thraustochytrids can use xylose specifically and no xylose metabolic pathway has been described in thraustochytrids.

The thraustochytrid strain T18 (T18) was isolated from Atlantic Canadian waters and produces large amounts of biomass containing up to 82% total fatty acids [23]. To unlock the biotechnology potential of this organism, we developed genetic engineering methods to create a T18 derivative strain able to efficiently convert hemicellulosic derived xylose into biomass and lipids.

Herein, we demonstrate the ability of wild-type T18 to use xylose. We also identify the presence of enzymes from the two xylose metabolism pathways in T18 by demonstrating the production of xylitol, a product of a xylose reductase, as well as the presence of an active xylose isomerase, a component of the second xylose metabolic pathway. To our knowledge, the presence of enzymes from two native xylose metabolism pathways in one organism has not previously been described. To improve xylose metabolism, we over-expressed the native xylose isomerase and a heterologous xylulose kinase in T18, increasing xylose usage and a reducing the production of xylitol. Enhanced xylose usage required the presence of multiple copies of the xylose isomerase and xylulose kinase genes, especially for growth on xylose as a sole carbon source.

## Methods

### Strains and plasmids

Thraustochytrid, *Escherichia coli*, and *Saccharomyces cerevisiae* strains used in this study are described in Table 1*. E. coli* and *S. cerevisiae* strains were propagated and transformed using standard techniques [24]. Thraustochytrid strains were grown at 25 °C in Windust light (WDL) media [1 g/L yeast extract, 4 g/L soy peptone, 2 g/L NaCl, 4 g/L MgSO_4_, 0.1 g/L CaCl_2_, 0.005 g/L FeCl_3_, 6.8 g/L (NH_4_)_2_SO_4_, 1.6 g/L KH_2_PO_4_, 1.75 g/L K_2_HPO_4_, trace elements (3 mg/L CuSO_4_5H_2_O, 3 mg/L ZnSO_4_7H_2_O, 1.5 mg/L Na_2_MoO_4_2H_2_O, 1.5 mg/L CoCl_2_6H_2_O, 1.5 mg/L MnCl_2_4H_2_O, and 1.5 mg/L NiSO_4_6H_2_O), and vitamins (30 μg/L vitamin B12, 30 μg/L biotin, and 0.006 g/L thiamin hydrochloride)] containing 60 g/L glucose.

**Table 1:** Strains used in this study

Organism	Strain name	Relevant characteristics	References
Thraustochytrid	T18	Wild-type thraustochytrid. ATCC PTA-6245	[23]
	XI 4	T18 transformed with pα-tub isohis. Homozygous knockout of an α-tubulin gene with *ble*-*2A*-*his*-*xi*. Zeocin-resistant	This study
	XI 6	T18 transformed with pα-tub isohis. Random insertion of *ble*-*2A*-*his*-*xi*. Zeocin-resistant	This study
	XI 8	T18 transformed with pα-tub isohis. Heterozygous knockout of an α-tubulin gene with *ble*-*2A*-*his*-*xi*. Zeocin-resistant	This study
	XI 16	T18 transformed with pα-tub isohis. Homozygous knockout of an α-tubulin gene with *ble*-*2A*-*his*-*xi* concatemer. Zeocin-resistant	This study
	XI-XK 1	XI 16 transformed with pJB47 (*E. coli xylB*). Zeocin and hygromycin-resistant	This study
	XI-XK 3	XI 16 transformed with pJB47 (*E. coli xylB*). Zeocin- and hygromycin-resistant	This study
	XI-XK 7	XI 16 transformed with pJB47 (*E. coli xylB*). Zeocin- and hygromycin-resistant	This study
	XB	T18 transformed with pJB13. Homozygous knockout of an α-tubulin gene with *ble*-*2A*-*xylB*. Zeocin-resistant	This study
*E. coli*	W3110	*F- mcrA mcrB IN*(*rrnD-rrnE*)*1 lambda*. ATCC^®^27325	ATCC
	BL21(DE3)pLysS	*F*- *ompT hsdSB*(*rB*–*mB*-) *gal dcm* (DE3) pLysS (CamR)	Bioline
	BL21 [pET23-XylA]	AmpR, CamR. pET23-XylA (W3110 histidine-tagged- *xylA*) in BL21(DE3)pLysS	This study
*S. cerevisiae*	INVSC1	MATa *his3*Δ*1 leu2 trp1*-*289 ura3*-*52*/MATα *his3*Δ*1 leu2 trp1*-*289 ura3*-*52*	Invitrogen
	INVSC1 [pYes2-isohis]	URA. Histidine-tagged T18 xylose isomerase under the control of the GAL1 promoter	This study

Plasmids and primers used in this study are described in Additional file 1: Tables S1, S2, respectively. The T18 xylose isomerase gene (1.32 kb), *xi*, was PCR amplified from genomic DNA using primers, AMJP1 and AMJP2. The PCR gene was inserted into pCR2.1-Topo (Invitrogen) via TOPO TA cloning (Invitrogen) resulting in the plasmid pTopoXyloIso.

To construct the N-terminal histidine-tagged xylose isomerase construct for expression in *E. coli*, the xylose isomerase gene was amplified from pTopoXyloIso with primers, AMJP15 and AMJP16. The amplified fragment was cloned into *Nco*I/*Bam*HI cut pET23der/MTF [25] resulting in plasmid pET23-isohis.

To express T18 xylose isomerase in yeast, the *Bgl*II/*Not*I fragment-containing His-tagged xylose isomerase from pET23-isohis was subcloned into *Bam*HI/*Not*I cut pYES2 (Invitrogen) resulting in plasmid pYES2-isohis.

The xylose isomerase gene (1.77 kb), *xylA*, from *E. coli* strain W3110 was amplified using primers AMJP11 and AMJP12. The amplified fragment was cloned into pCR2.1-Topo via TOPO TA-cloning resulting in pTopoXylA.

An N-terminal histidine-tagged version of *xylA* was constructed by amplifying the xylose isomerase gene from pTopoXylA using primers AMJP12 and AMJP20 and then cloning it into *Nco*I/*Bam*HI cut pET23der/MTF resulting in pET23-XylAhis.

To express additional copies of T18 xylose isomerase in T18, the His-tagged xylose isomerase gene from pYES2-isohis was amplified with AMJP39 and AMJP40 and cloned into *Sbf*I/*Not*I of pJB5 to create pα-tub isohis. The insert in pα-tub isohis thus contains an α-tubulin promoter (1.01 kb) from T18 driving expression of *ble* (for selection on zeocin) linked by a self-cleaving 2A peptide (GSGATNFSLLKQAGDVEENPGP) from porcine teschovirus-1 [26] to the His-tagged T18 xylose isomerase gene (1.37 kb) followed by an α-tubulin terminator (1.02 kb) from T18.

To express a xylulose kinase in T18, the *E. coli* W3110 *xylB* gene (1.46 kb) was codon optimized for expression in T18 and commercially synthesized (GENEWIZ). The codon-optimized *xylB* gene was inserted immediately 3′ to an α-tubulin promoter linked to *ble*-2A by subcloning into *Sbf*I/*Not*I cut pJB3, resulting in pJB13. Finally, *ble* was replaced by *aph7* (for selection with hygromycin), which was amplified from pChlamy_3 (Invitrogen) using JBo28 and JBo29 and then cloned into *Xba*I/*Kpn*I cut pJB13, resulting in pJB47.

### Transcriptomics analysis of T18 and xylitol identification

Three parallel batch fermentations were performed with T18 in WDL media containing 60 g/L glucose and 10 g/L xylose. Samples were taken during the fermentation and the sugar concentration in the culture supernatants was analyzed by HPLC with refractive index (RI) detection. Transcriptomics analysis was performed on methanol-dry-ice flash frozen cell pellets by a third-party laboratory (Metabolomic Discoveries GmbH) using microarrays. The identification of xylitol in the culture supernatant through mass spectrometry was completed by this external laboratory as well as by the National Research Council (NRC) Canada.

### Protein over-expression and purification of T18 xylose isomerase and *E. coli* XylA and XylB

His-tagged T18 xylose isomerase was over-expressed in *S. cerevisiae* strain INVSc1 (Invitrogen) by growing the strain in SC minimal medium-lacking uracil and containing 2% (w/v) galactose according to the manufacturer’s specifications. His-tagged XylA and XylB were over-expressed in BL21(DE3)pLysS *E. coli* through IPTG induction. The enzyme purification was performed by metal chelation using standard protocols with 50 mM HEPES buffer pH 7.4, 0.5 M NaCl and increasing amounts of imidazole. Protein concentration was determined using the Bio-Rad Bradford Assay and bovine serum albumin as a standard.

### Transformation of T18

Biolistic transformation of T18 was performed as previously described [27]. Briefly, DNA plasmids were linearized with *Eco*RI or *Hin*dIII before transformation. One millilitre T18 cultures grown to an OD_600nm_ of 1 were spread on WDL agar plates containing no antibiotics. The cells were bombarded with DNA coated gold particles (0.6 μm diameter) in a Biolistic PDS-1000/He system (Bio-Rad). The bombardment conditions included using 2.5 μg DNA, 1100 or 1350 psi rupture disks, a target distance of 3 or 6 cm, and a Hg vacuum between 25 and 27.5 inches. Cells were recovered overnight at 25 °C, collected, and spread on WDL plates containing the selection antibiotic. Clones transformed with *ble* constructs were selected on WDL agar plates containing 250 μg/mL zeocin. *aph7*-containing transformants were selected on WDL agar plates containing 400 μg/mL hygromycin. The presence of the transgenes was confirmed via PCR screening and Southern blot analysis.

### Southern blot analysis

Southern blotting was performed using the digoxigenin-labeling method and detection with anti-digoxigenin-alkaline phosphatase-CSPD (disodium 3-(4-methoxyspiro{1,2-dioxetane-3,2′-(5′-chloro)tricyclo [3.3.1.1^3,7^]decan}-4-yl)phenyl phosphate) substrate as specified by the manufacturer (Roche). Genomic DNA was extracted from T18 transformants using a commercial microbial DNA isolation kit (MoBio Laboratories) and cut with *Hin*dIII and *Sca*I or *Sca*I and *Sbf*I.

### qPCR

The copy numbers of genes *xi*, *ble*, and *aph7* were determined by quantitative PCR using SensiFast SYBR Lo-Rox (Bioline) with an Agilent thermocycler according to the manufacturer’s instructions. Primers, described in Additional file 1: Table S3, were designed to yield products 75–150 bp in length and assayed for amplification efficiency. The primers for the internal reference gene, *actin* or *GAPDH*, used for quantification of a particular gene were matched to this amplification efficiency (between 89.9 and 110%). All reactions were performed in triplicate. Template genomic DNA was isolated from T18 transformants as described above.

### Preparation of soluble T18 cell extracts for enzyme assays and Western blotting

T18 cell pellets were resuspended in ice-cold “breaking buffer” (50 mM HEPES pH 7.4, 10 mM MgCl_2_) and acid-washed glass beads (Sigma) were added to the suspension. For Western blotting, 1X protease inhibitor cocktail (Cell Signaling Technology 5871S) was added to the buffer. The cells were lysed by vigorous vortexing (five 30 s intervals with a minute incubation on ice). The suspension was centrifuged at 12,000×*g* for 10 min to remove unlysed cells and glass beads. Total protein concentration of the supernatants was measured using the Bio-Rad protein determination assay. Supernatants from each transformant were then diluted at an equal protein concentration in breaking buffer for enzyme assays.

### Enzyme assays with purified enzymes and T18 cell extracts

Typical enzymatic reactions contained approximately 0.75 μg/μL xylose or xylulose, 5 mM MgATP, 10 mM MgCl_2_, 50 mM HEPES (pH 7.4) [28], and increasing amounts of either purified xylose isomerases, xylulose kinase, or cell extracts. After overnight incubation at either 50 °C or 30 °C, the enzymatic activity was stopped by boiling for 5 min and reactions were analyzed by HPLC on an Aminex HPX-87H 300 × 7.8 mm column (Bio-Rad) with RI detection.

### Western blotting

SDS-PAGE analysis and Western blotting with mouse anti-histidine antibody (Bio-Rad #6200203, 1:10 000), rabbit anti-2A antibody (Sigma-Aldrich ABS31, 1:10 000), anti-mouse antibody (Cedarlane ab8224, 1:3000), and goat anti-rabbit antibody conjugated to Horseradish Peroxidase (Bio-Rad #1662408, 1:2000) were done according to standard procedures. Protein extracts were incubated for 30 min at 37 °C or 5 min at 100 °C prior separation by SDS-PAGE.

### Xylose depletion assays

T18 transformants were grown for 3 days in WDL broth at 25 °C. Xylose depletion assays were performed in Media 2 minimal media (9 g/L NaCl, 4 g/L MgSO_4_, 0.1 g/L CaCl_2_, 0.005 g/L FeCl_3_, 20 g/L (NH_4_)_2_SO_4_, 0.86 g/L KH_2_PO_4_, trace elements, and vitamins) containing 20 g/L glucose and 50 g/L xylose. Cells were gently washed in 0.85% NaCl and then added to Media 2 to a final OD_600nm_ of 0.05. Four millilitre samples were taken over the course of the experiment to determine the amount of biomass produced as well as the amount of sugar remaining in the media. Sugar concentrations in the media were determined by HPLC on an Aminex HPX-87H column (Bio-Rad) with RI detection. Biomass concentration was determined by weighing dried cell pellets.

Xylose depletion assays testing growth in the presence of xylose only were done with 50 g/L xylose in Media 2 minimal media and WDL broth. Cultures were inoculated to a final OD_600nm_ of 0.25.

The amount of xylose used and biomass produced by the transformants were compared to that of the wild type using Student’s *t* tests.

### Fermentation of T18 strain XI-XK 7

Fermentations were carried out in 7 L vessels. Media was batched with 3 L WDL media containing 2% glucose (w/v) and 5% xylose (w/v). The culture was seeded with 300 mL of a flask culture grown in WDL media with 6% glucose (w/v). The culture was stirred with 2–6 bladed Rushton impellers with a diameter of 54 cm at 2.2 m/s. Atmospheric air was sparged into the vessel at a rate of 1.0 VVM. The pH was maintained at 5.75 with 5 M NaOH and the temperature was maintained at 28 °C throughout. Dissolved oxygen was monitored throughout the fermentation as an indication of glucose consumption. Samples were taken during the fermentation to determine the concentrations of glucose, xylose, and xylitol by HPLC. After the initial batched glucose was exhausted, as indicated by a sudden spike in dissolved oxygen, samples were taken at 2 h intervals for 4–6 h. The cultures were then fed with 75% (w/v) glucose at a rate of 10 g/L-h for 16 h after which two additional glucose starvation/feeding cycles were initiated. Fermentations lasted 98 h. Dried cell weight was also determined using freeze-dried washed cell pellets. The lipid content of the cells was determined by the GC analysis of FAMEs as described previously [29].

## Results

### Wild-type T18 uptakes xylose and produces xylitol

**Fig. 2:**
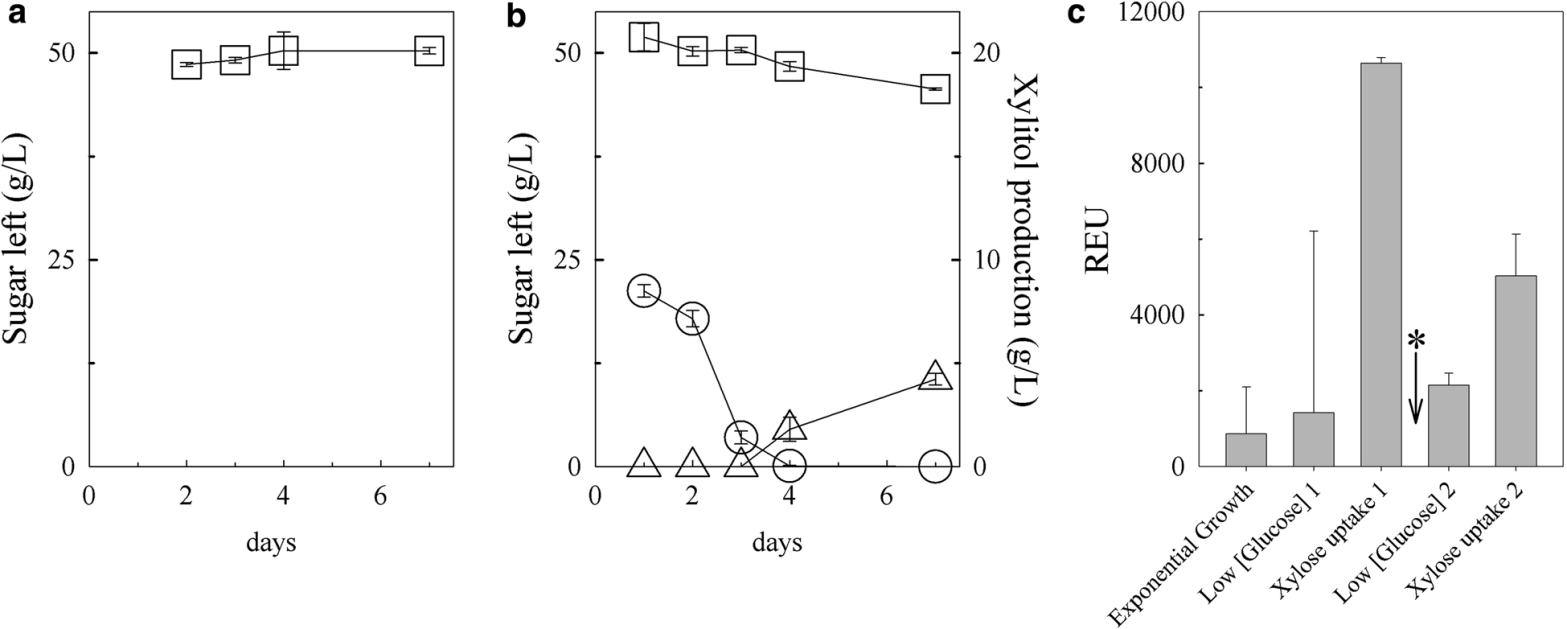
Sugar utilization and xylose isomerase transcription by wild type grown in xylose with and without glucose. **a** The amount of xylose (squares) remaining in the culture supernatant when grown in the absence of glucose. **b** Amount of glucose (circles), xylose, and xylitol (triangles) present in the culture supernatant when grown in the presence of both sugars. Error bars represent standard deviation from triplicates. **c** Cultures were batched in glucose and xylose then re-fed glucose after the first xylose uptake period as indicated by the asterisk. Error bars represent standard deviation from three fermentations

Since thraustochytrids are found associated with healthy and decomposing plant matter, we hypothesized that they may have the ability to consume sugars present in hemicellulosic material. The ability of wild-type T18 to consume xylose was investigated by growing it in minimal media containing xylose with and without glucose, and measuring the amount of each sugar remaining in the supernatant by HPLC at different time points (Fig. 2a, b). In the presence of xylose alone, no significant consumption of xylose was observed (Fig. 2a). In contrast, when grown in the presence of both sugars, glucose was the primary sugar consumed on days 1 and 2. Only when glucose levels were low (day 3) did the concentration of xylose in the medium start decreasing, with most of the decrease occurring between days 4 and 7 (Fig. 2b). Analysis of the chromatographs revealed that, by day 7, an additional peak migrating at 9.2 min was detected (Additional file 2: Figure S1A). The peak was identified as xylitol by comparing the migration of this unknown sugar with known standards (Additional file 2: Figure S1B) and mass spectrometry (Additional file 2: Figure S1C). The decrease of xylose in the media suggests that T18 encodes a sugar transporter(s) capable of uptaking xylose, whereas the production of xylitol suggests that a gene encoding for a xylose reductase is present in T18. Of the ~ 13 g/L xylose uptaken by T18, 76% was converted to xylitol and released in the supernatant. It is not known whether the remaining xylose was metabolized beyond xylitol via a xylitol dehydrogenase or whether another xylose metabolic pathway is present in T18. The presence of xylitol in the media, however, suggested that shuttling xylitol through to the xylitol dehydrogenase is limited, perhaps, by a co-factor imbalance as shown in other organisms [12] or due to the absence of a functional xylitol dehydrogenase in T18.

### T18 encodes a putative xylose isomerase which is highly expressed during xylose consumption

Efforts to identify T18 gene products involved in xylose metabolism in T18 by bioinformatics analysis of the genome revealed a 1.32 kb open-reading frame encoding a protein of 439 amino acids and a calculated molecular weight of 49.5 kDa. The encoded protein has 48–60% identity to known xylose isomerases proteins and contains a conserved TIM (triosephosphate isomerase) phosphate-binding superfamily domain common to these enzymes [30].

To further examine whether the putative T18 xylose isomerase gene is involved in xylose metabolism, transcript levels of this gene were measured in microarrays during glucose and xylose consumption phases (Fig. 2c). The level of the transcript was relatively low [< 1000 Relative Expression Units (REU)] during exponential phase when glucose was being consumed. When the glucose concentration was very low and the amount of xylose in the supernatant started to decrease (Xylose consumption 1), the level of the putative xylose isomerase transcript increased. Refeeding the culture with glucose led to a decrease in the quantity of the transcript. Once this re-fed glucose was consumed and xylose consumption resumed (Xylose consumption 2), the transcript levels increased again. The link between the gene expression of this putative xylose isomerase and xylose consumption further supported the examination of the potential xylose isomerase activity of the protein encoded by this gene.

### The putative T18 xylose isomerase has xylose isomerase activity in vitro

To examine the enzymatic properties of the putative xylose isomerase, we cloned the gene from T18 under a *GAL1* promoter with an N-terminal histidine tag to allow expression in yeast and purification of the protein (with a calculated molecular weight of 50.8 kDa with the tag) by metal chelation. Despite optimizing the imidazole concentrations for elution, fractions containing the putative T18 xylose isomerase (migrating at approximately 52 kDa) also contained a prominent co-eluting ~ 39 kDa protein (Additional file 2: Figure S2). Based on band intensity, the T18 protein is calculated to be 13% of the fraction. Despite binding to the Ni2 + NTA resin and confirmation that the histidine tag was in frame with the putative xylose isomerase gene, we could not detect a histidine tag on the purified protein fraction by Western immunoblot. This may be due to the known variability issues with immunodetection of His-tagged proteins, including, for example, the accessibility of the antibody to his tag due to the protein structure, or even the temperature at which the sample is heated prior loaded on the gel [31]. As such, a fraction containing mainly the co-eluting 39 kDa protein (Additional file 2: Figure S2, lane 3) was used as negative control in the protein activity assays.

Protein activity assays on xylose and xylulose were done with fractions containing the putative xylose isomerase protein. As a positive control, a known xylose isomerase, XylA, from *E. coli* was also cloned with a N-terminal histidine tag, and over-expressed and purified from *E. coli*. Xylose isomerases catalyze the interconversion of xylose and xylulose; however, at equilibrium, the enzymes favour xylulose as substrate [32] (Fig. 1). As such, the proteins were incubated with each sugar and the amount of substrate conversion was determined via HPLC. The initial assays incubated at 30 °C, the optimal temperature for XylA (Additional file 2: Figure S3A), showed that the purified T18 protein could indeed interconvert xylose and xylulose (data not shown) which was further evidence that the purified protein is a functional xylose isomerase. As the activity of T18 enzyme on xylose, specifically, was best at higher temperatures (Additional file 2: Figure S3B), further experiments with T18 xylose isomerase were done at 50 °C.

Dose dependence was confirmed by incubating increasing amounts of XylA, T18 xylose isomerase, or the negative control fraction containing mainly the co-eluting protein with a fixed amount of xylose or xylulose (Additional file 2: Figure S4). Both XylA and T18 xylose isomerase showed increased activity on both sugars as the protein concentration increased. In contrast, very little xylose was made when xylulose was incubated with total protein containing the 39 kDa protein and undetected amounts of T18 xylose isomerase, confirming that the 52 kDa protein is responsible for the xylose isomerase activity. As the xylose isomerase made up 13% of the total protein, the similar levels of activity on xylulose between the two enzymes despite the differences in concentration indicate that T18 xylose isomerase has a much higher isomerase activity than XylA in these assays.

Some xylose isomerases from other organisms have affinity for additional sugars such as glucose and fructose [33]; however, no activity was seen when T18 xylose isomerase was incubated with either glucose or fructose under these conditions (data not shown).

### Homologous recombination of the xylose isomerase in thraustochytrid T18 results in transformants with varying copy numbers

The confirmed xylose isomerase activity in T18 as well as the production of xylitol when grown in the presence of xylose indicated that T18 encodes for enzymes from two xylose metabolism pathways: a xylose reductase/xylitol dehydrogenase pathway as well as a xylose isomerase pathway. Because xylose reductase/xylitol dehydrogenase pathways in other organisms can be affected by co-factor imbalance, resulting in the release of xylitol into the media, the non-co-factor-dependent xylose isomerase pathway is often considered to be optimal for efficient xylose usage [12]. Reduced levels of xylose isomerase transcript in the presence of glucose suggest that xylose isomerase expression is regulated in T18. The effect of constitutively expressing the xylose isomerase on xylose utilization was examined by cloning the His-tagged T18 xylose isomerase gene (*xi*) linked to a zeocin selection marker (*ble*) by a self-cleaving 2A peptide encoding sequence under the control of an α-tubulin promoter and transforming the resulting *ble*-*2A*-*his*-*xi* construct into T18.

Four representative transformants (XI 4, XI 6, XI 8, and XI 16) were examined for homologous recombination at an α-tubulin locus by Southern blotting using an α-tubulin locus-specific probe (Additional file 2: Figure S5A) and a *ble*-specific probe (Additional file 2: Figure S5B). Based on the Southern blot analysis and evidence that some thraustochytrids are diploids [27, 34], XI 6 was determined to have the transgenes randomly inserted in its genome, XI 8 was determined to be a heterozygote, with the transgene replacing one of the two copies of the α-tubulin at the targeted locus, and XI 4 and XI 16 were determined  to be homozygotes. Interestingly, in XI 16, the much larger (~ 23 kb) band at the α-tubulin locus indicates the presence of a construct larger than a single copy of the *ble*-*2A*-*his*-*xi* construct seen in XI 4 and XI 8.

**Fig. 3:**
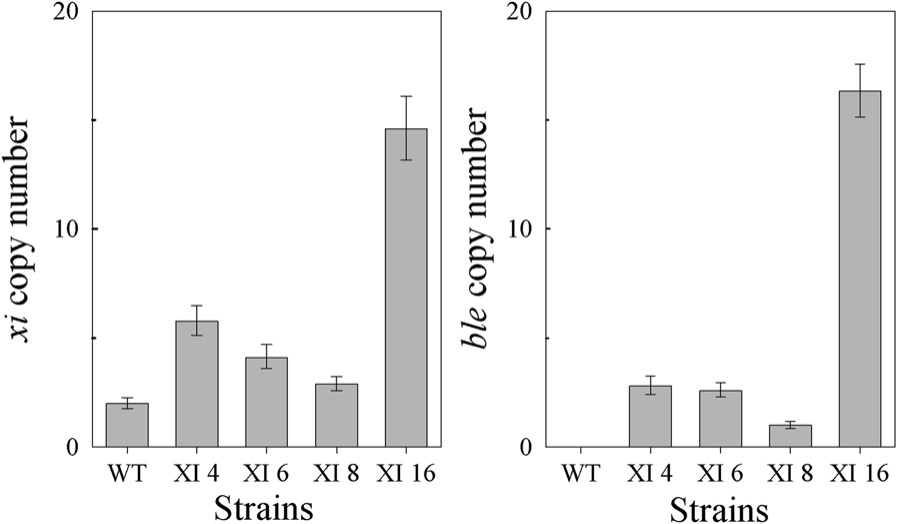
qPCR analysis of wild-type (WT) and XI transformants strains. The gene copy number of **a** the endogenous and His-tagged transgenic *xi* genes and **b** the transgenic *ble* gene were measured. Experiments were done in triplicate. Error bars represent the higher and lower relative quantity limits

The four transformants were further characterized by qPCR to estimate the copy number of the transgenes. The amount of DNA between strains was normalized using the *GAPDH* gene. qPCR analysis using primers specific to the native xylose isomerase gene (Fig. 3a) revealed that XI 8 has one additional transgenic copy of the xylose isomerase gene compared to the WT control strain which has two copies of the xylose isomerase gene, XI 6 has two transgenic copies, XI 4 has four transgenic copies, and XI 16 has at least 12 transgenic copies (Fig. 3a). qPCR with primers specific to *ble* and DNA concentration normalization based on the actin gene confirmed the xylose isomerase results (Fig. 3b).

### Increased xylose isomerase copy number correlates with increased xylose isomerase activity

2A sequences are often used to introduce multiple proteins on a single transcript, whereby peptide cleavage occurs through ribosome “skipping” at the 2A site [35]. 2A cleavage of Ble-2A and His-XI from the *ble*-*2A*-*his*-*xi* construct in T18, albeit, not very efficient, was confirmed by Western blot (Additional file 2: Figure S6).

To determine whether the differences in xylose isomerase gene copy number between the four *ble*-*2A*-*his*-*xi* transformants (XI 4, XI 6, XI 8, and XI 16) and wild type correlated with differences in xylose isomerase activity between the strains, increasing amounts of cell extract from these strains were incubated with a set amount of xylose or xylulose, and the amount of substrate conversion was assessed by HPLC.

A low level of xylose isomerase activity was observed in wild-type cell extract with 0.8 μg/μL of wild-type cell extract required to make 0.11 μg/μL xylulose and 0.29 μg/μL xylose from xylose and xylulose, respectively (Additional file 2: Figure S7A and B). In contrast, protein extracts from all four transformants had higher isomerase activity than protein extract from wild type on both sugars over the protein concentration range tested.

Protein dose dependence was observed with protein extracts from the four transformant strains, demonstrating that the histidine-tagged xylose isomerase is functional in vitro. Comparing xylose isomerase activities at the same total protein concentration revealed that the increased copy number of the His-tagged xylose isomerase gene in the transformants correlated with a corresponding increase in xylose isomerase activity (Additional file 2: Figure S7A and B). For example, reactions with 0.2 μg/μL, protein extracts from XI 8, which harbors an extra xylose isomerase copy, had up to 2.3× higher isomerase activity compared to wild type. Similarly, compared to the activity from wild-type protein extracts, protein extracts from XI 6, with its two additional xylose isomerase copies, had 2.7× times more activity; protein extracts from XI 4 with its four additional copies had up to 3.6× more activity; and protein extracts from XI 16 with its 12 extra copies resulted in up to 4.7× more activity.

### Multiple copies of T18 xylose isomerase are required to increase xylose consumption and reduce xylitol production

**Fig. 4:**
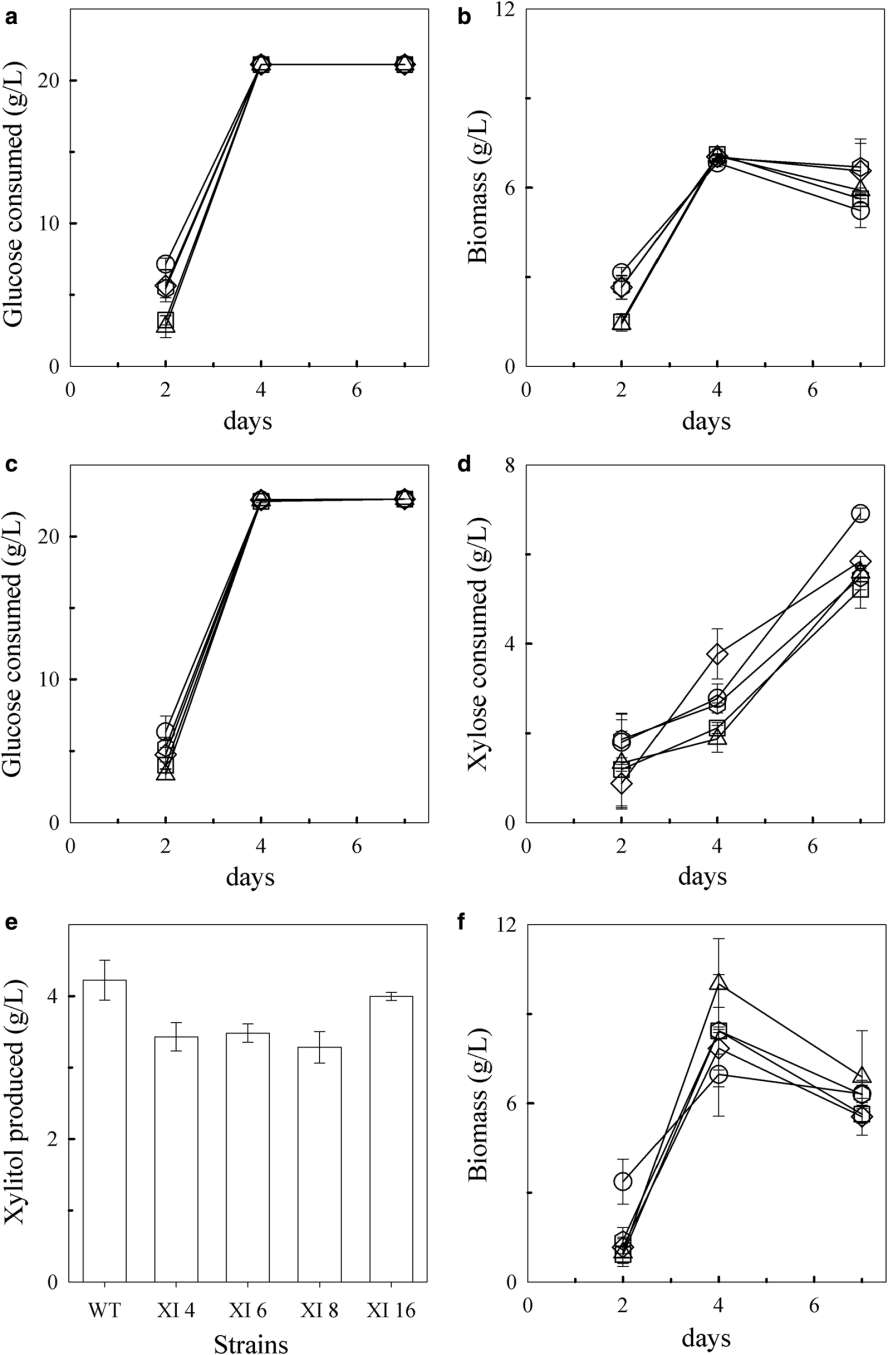
Wild-type (WT) and XI transformants growth in minimal-media containing glucose with and without xylose. Glucose consumption (**a**) and biomass accumulation (**b**) in media containing glucose alone. Consumption of glucose (**c**) and xylose (**d**), xylitol production by day 7 (**e**), and biomass accumulation (**f**) in media containing glucose and xylose. The experiments were done in triplicate. Error bars represent standard deviations. Symbols: diamonds, wild type; squares, XI 4; triangles, XI 6; hexagons, XI 8; and circles, XI 16

The ability of the four xylose isomerase transformants to grow in minimal media containing glucose with and without xylose was examined. The amount of sugar remaining in the supernatant over time was determined by HPLC. In both media, glucose was depleted by day 4 in all the strains (Fig. 4a, c). A preference for glucose over xylose was still observed in the xylose isomerase transformants with xylose only noticeably consumed when glucose levels were low or absent (Fig. 4c, d). Glucose and xylose consumption in the xylose isomerase transformants XI 4, XI 6, and XI 8 was similar to wild type by day 7 (Fig. 4a, b) despite having higher xylose isomerase copies. In contrast, XI 16 consumed glucose similarly to wild type but consumed slightly, but statistically significantly (*p* value = 0.006), more xylose (6.9 ± 0.13 g/L) than wild-type (5.9 ± 0.11 g/L) by day 7 (Fig. 4c, d). Presumably, co-factor imbalance is still a factor as the transformants still released xylitol in the supernatant (Fig. 4e). However, while wild type secreted 4.2 ± 0.28 g/L xylitol (representing 72% of the xylose consumed), XI 16 secreted 4.0 ± 0.06 g/L which represents 58% of the xylose consumed. This relative reduction in xylitol produced from xylose consumed suggests that more of the xylose taken up was shuttled away from the xylitol pathway in this transformant. Similarly, on average, the amount of xylitol produced relative to the amount of xylose consumed by the other XI transformants was less than wild type which further suggests that additional xylose isomerase copies provide a slight benefit to these strains.

The average biomass of the transformants and the wild-type strains initially increased by day 4, but then decreased significantly by day 7 in both media (Fig. 4b, f); however, that decrease was more significant (*p* value = 0.005) in media containing xylose, even though, by day 7, xylose is the predominant carbon being consumed (Fig. 4f). While the average biomass decrease in media containing glucose alone (1.0 ± 0.8 g/L) may be due to cells dying in the absence of any carbon available, the reason for the average decrease in media containing xylose (2.2 ± 1.3 g/L) during xylose consumption is unknown but may be related to the toxic accumulation of xylitol in the media [36]. Nonetheless, despite consuming more xylose and producing less xylitol than wild-type, transformant XI 16 did not make significantly more biomass by day 7.

### Transformation of the xylose isomerase over-expressing strain with xylB results in genomic rearrangement of the original *ble-2A-his-xi* insert

The presence of a native xylulose kinase in T18 was inferred by the contribution of xylose to a consistently higher biomass accumulation in both wild-type and XI strains grown in the presence of both glucose and xylose compared to cells grown glucose alone on day 4 (Fig. 4b, f). The native xylulose kinase gene, however, could not easily be identified in the T18 genome. Cell extract assays on the wild-type and xylose isomerase transformants with xylulose did not reveal high xylulose kinase activity (data not shown), suggesting that the native xylulose kinase is not expressed at high levels or is not active under the experimental conditions used. Furthermore, the continued presence of xylitol in the media of the transformants suggests that co-factor imbalance has not been resolved by over-expression of the xylose isomerase alone. We hypothesized that increasing the levels of both the xylose isomerase and a xylulose kinase in T18 would divert xylose away from the T18 xylose reductase and xylitol dehydrogenase pathway, and result in enhanced xylose utilization. The *E. coli* xylulose kinase gene, *xylB*, was codon optimized for expression in T18 and cloned with a hygromycin resistance gene (*aph7*) under the control of an α-tubulin promoter and terminator. The *aph7*-*2A*-*xylB* construct was transformed into XI 16. Preliminary in vitro experiments combining purified T18 xylose isomerase, *E. coli* XylB, and xylose resulted in a significant decrease in the amount of xylose, suggesting that there was a conversion to xylulose-5-phosphate and that these enzymes can work in concert (data not shown).

A number of XI 16 [*aph7*-*2A*-*xylB*] transformants were obtained and screened by PCR for gene insertion. Three representative transformants, XI-XK 1, XI-XK 3, and XI-XK 7, were selected for further analysis by Southern blot with probes specific for the α-tubulin loci, *ble*, and *aph7* (Additional file 2: Figure S8). Changes in banding patterns with both the α-tubulin loci and *ble* probes were observed in all the three transformants compared to the parental strain, XI 16, indicating rearrangement in the *ble*-*2A*-*his*-*xi* construct. The mechanisms for these changes are not well understood.

**Fig. 5:**
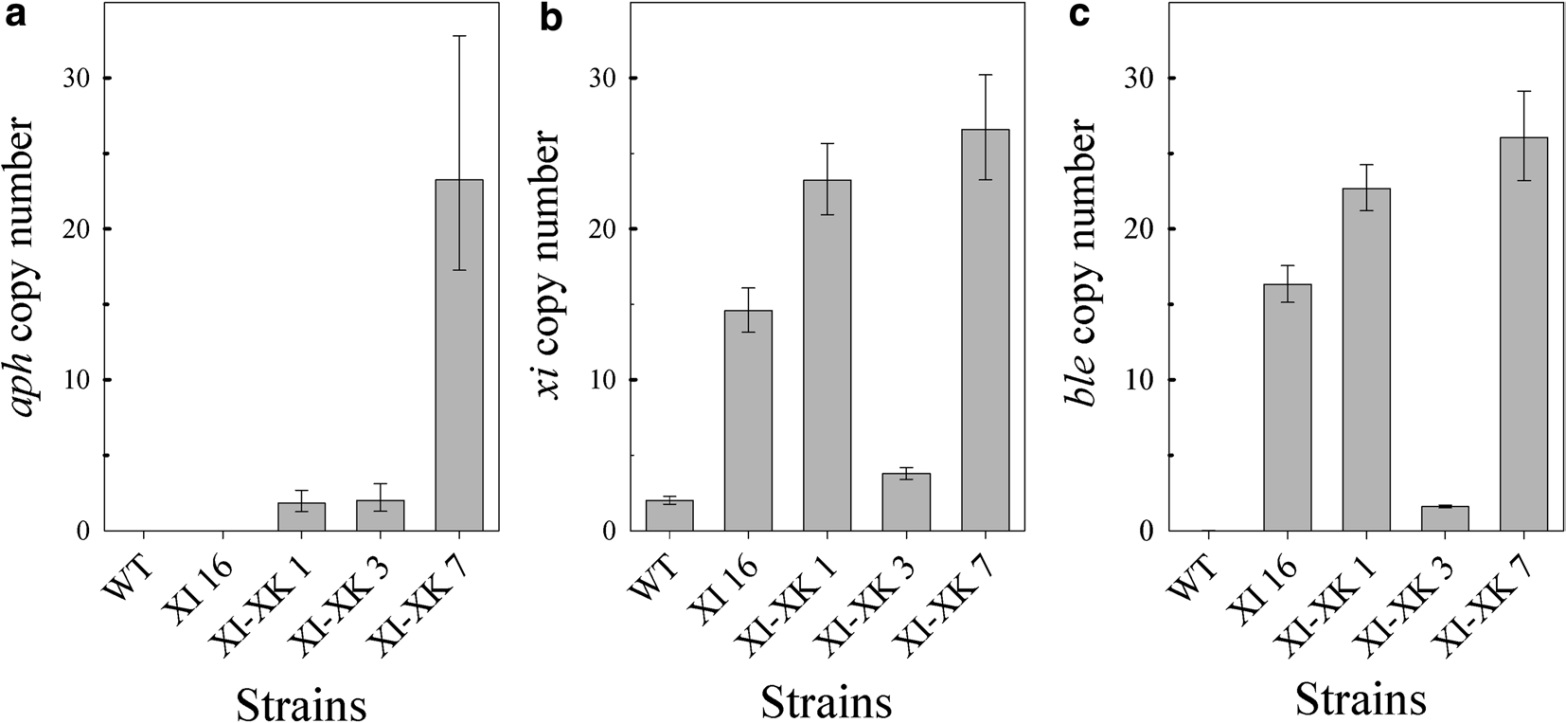
qPCR analysis of wild-type (WT) and XI-XK transformants strains. Gene copy numbers were measured for **a** *aph7*, **b** the native and His-tagged xylose isomerase, and **c** *ble*. Experiments were done in triplicate. Error bars represent the higher and lower relative quantity limits

To estimate the gene copy number for both the xylose isomerase and xylulose kinase genes in these transformants, qPCR analysis was performed on genomic DNA using primers specific to *aph7*, *ble*, and *xi*. Using *actin* as an internal control, transformants XI-XK 1 and XI-XK 3 were shown to have at least two copies of *aph7* and presumably *xylB* as these genes are genetically linked. Strikingly, transformant XI-XK 7 had approximately 23 copies of the gene (Fig. 5a). qPCR performed with xylose isomerase specific primers revealed that XI-XK 3 has only four copies of the xylose isomerase genes, indicating that upon transformation with the *aph7*-*2A*-*xylB* construct, at least eight copies of the xylose isomerase gene were lost from the parental strain (Fig. 5b). Most surprising, however, was the 8–12 additional xylose isomerase gene copies detected in transformants XI-XK 1 and XI-XK 7 compared to the parental strain. These results were confirmed using primers specific to *ble* which is genetically linked to the histidine-tagged xylose isomerase gene and showed a similar decrease from parental copy number in XI-XK 3 and increases in copy number in XI-XK 1 and XI-XK 7 (Fig. 5c). The mechanism for gene loss and gain in these strains is still not understood.

### Increased expression of both xylose isomerase and xylulose kinase in T18 correlates with increased conversion of xylose to xylulose-5-phosphate in vitro

To assess how the variations in gene copy number for xylose isomerase and xylulose kinase genes in transformants XI-XK 1, XI-XK 3, and XI-XK 7 affect the activity of these enzymes in vitro, we performed cell extract activity assays using xylose and xylulose as substrate. These assays were performed at 30 °C as XylB is active at this temperature [37], and substrate conversion was determined by HPLC. As a positive control for xylulose kinase activity, *xylB* was transformed into wild-type T18. Transformant XB, a homozygous α-tubulin knockout with 23.6 *ble*-*2A*-*xylB* copies (Table 1 and 2, and Additional file 2: Figure S9), was chosen based on its high xylulose kinase activity.

**Table 2:** Gene copy number of wild-type and transformants and protein activity on xylose and xylulose

Strain	Gene copy number^a^			% sugar used/μg/μL sugar left^b^		μg/μL xylulose-5-phosphate^c^
	*ble* ^d^	*xi*	*aph7*	Xylose	Xylulose	
WT	–	2.0	–	− 0.21 ± 0.28/0.55 ± 0.002	1.59 ± 0.58/0.62 ± 0.004	0.000 ± 0.004
XI 16	16.3	14.6	–	0.26 ± 0.29/0.55 ± 0.002	12.9 ± 0.26/0.55 ± 0.002	0.000 ± 0.001
XI-XK 1	22.7	23.2	1.8	10.2 ± 0.21/0.50 ± 0.002	42.5 ± 0.38/0.36 ± 0.002	0.035 ± 0.001
XI-XK 3	1.6	3.8	2.0	0.67 ± 0.13/0.55 ± 0.001	8.88 ± 0.67/0.58 ± 0.004	0.001 ± 0.004
XI-XK 7	26.1	26.6	23.2	26.2 ± 0.32/0.41 ± 0.002	92.1 ± 0.47/0.05 ± 0.003	0.404 ± 0.003
XB	23.6	2.0	–	0.14 ± 0.34/0.55 ± 0.002	97.8 ± 3.9/0.01 ± 0.025	0.619 ± 0.026

*N.D.* not determined

^a^Gene copy numbers were determined by qPCR

^b^Cell extracts were done with set amounts of xylose (0.55 μg/μL) and xylulose (0.63 μg/μL) and the amount of sugar detected was determined by HPLC. The amount of sugar converted or detected following incubation with 0.08 μg/μL total protein extract is shown

^c^Xylulose-5-phosphate produced with 0.08 μg/μL total protein was calculated by deducting the total amount of xylose and xylulose detected in these reactions from the known starting amount of xylulose

^d^*ble* values in the XB transformant indicate the gene copies of *ble*-*2A*-*xylB*, whereas, in the XI and XI-XK transformants, it indicates gene copies of *ble*-*2A*-*his*-*xi*

Under these conditions, changes in gene copy of the xylose isomerase and xylulose kinase in the transformants were once again shown to correlate with increases and decreases in enzyme activity (Additional file 2: Figure S10, Table 2). For example, XI-XK 3 protein extracts had a reduction in xylose isomerase activity compared to the parental strain XI 16 as expected with the decrease in *ble*-*2A*-*his*-*xi* copies in this strain (Additional file 2: Figure S10C, Table 2). Similarly, an increase in xylose isomerase activity over the parental strain in protein extracts from XI-XK 1 and XI-XK 7 also correlated with the increased *ble*-*2A*-*his*-*xi* copies in these strains. Little, if any, xylulose kinase activity was observed with protein extracts from the wild-type and XI 16 strains under these conditions (Additional file 2: Figure S10D, Table 2). Despite having two copies of *aph7*-*2A*-*xylB*, there was little xylulose kinase activity observed in protein extracts from XI-XK 3, whereas the two *aph7*-*2A*-*xylB* copies in XI-XK 1 only resulted in 0.035 μg/μL xylulose converted to xylulose-5-phosphate at 0.08 μg/μL total protein (Table 2). On the other hand, the high *aph7*-*2A*-*xylB* copies in XI-XK 7 correlated well with significant increases in xylulose kinase activity compared to the parental strain, XI 16 (Table 2).

### Transformants over-expressing both xylose isomerase and xylulose kinase consumed significantly more xylose and made significantly less xylitol

To assess the ability of transformants XI-XK 1, XI-XK 3, and XI-XK 7 to consume and metabolize xylose through the engineered xylose isomerase/xylulose kinase pathway, the strains were grown in minimal media containing glucose (20 g/L) with and without xylose (50 g/L). Sugar consumption and xylitol production were monitored by HPLC.

**Fig. 6:**
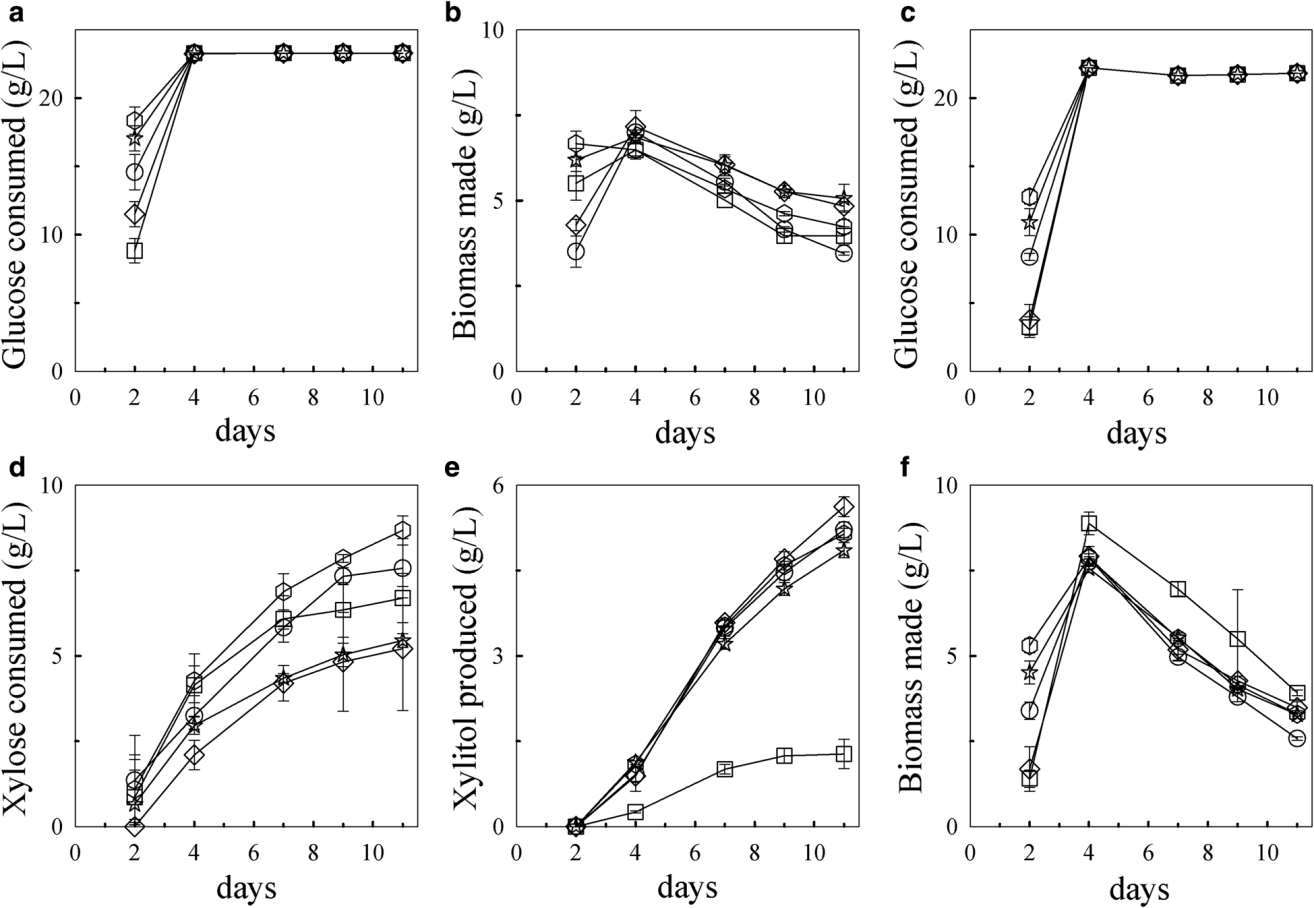
Wild-type and XI-XK transformants growth in minimal media containing glucose with and without xylose. Glucose consumption (**a**) and biomass accumulation (**b**) in media containing glucose alone. Consumption of glucose (**c**) and xylose (**d**), xylitol production (**e**), and biomass accumulation (**f**) in media containing glucose and xylose. The experiments were done in triplicate. Error bars represent standard deviations. Symbols: diamonds, WT; circles, XI 16; triangles, XB; hexagons, XI-XK 1; stars, XI-XK 3; squares, XI-XK 7

In both media, the glucose was depleted by day 4 (Fig. 6a, c). The wild-type preference for glucose over xylose when both sugars are present was not significantly altered in these transformants. Glucose was still consumed first (Fig. 6c) and xylose was consumed only when glucose levels were low or depleted (Fig. 6d). Under these conditions, wild type consumed 5.2 ± 1.8 g/L xylose, with most (5.6 ± 0.17 g/L) released in the media as xylitol by day 11 (Fig. 6e). The parental strain, XI 16, consumed 7.6 ± 0.87 g/L xylose and converted statistically significantly (*p* value = 0.012) less of it to xylitol (5.2 ± 0.01 g/L) than wild type. Despite having increased xylose isomerase gene copies over its parent, XI-XK 1 consumed statistically similar amounts of xylose (8.7 ± 0.43 g/L) and produced similar amounts of xylitol (5.1 ± 0.10 g/L) as the parent. In contrast, XI-XK 3 with its loss in xylose isomerase gene copy had lower xylose consumption (5.5 ± 0.20 g/L) than the parental strain and similar xylose consumption to wild type. The presence of both xylose isomerase and xylulose kinase in this transformant, however, correlated with a statistically significant reduction (*p* value = 0.004) in xylitol production (4.9 ± 0.13 g/L) compared to wild type. Transformant XI-XK 7 consumed a similar amount of xylose (6.7 ± 0.72 g/L) as the parental strain but made significantly less xylitol (1.3 ± 0.26 g/L) than all of the strains. This efficient utilization of xylose by transformant XI-XK 7 resulted in increased biomass relative to the other transformants after day 2 (Fig. 6f). Although the initial growth of XI-XK 7 was slower than all the strains, by day 4, its biomass surpassed the other strains and remained higher over the course of the experiment.

 Of note are the differences in the biomass production by all the strains when grown in the presence of glucose and xylose versus when grown in the presence of glucose alone. When grown in the presence of glucose and xylose, the biomass of all the strains peaked on day 4 (average 8.0 ± 0.5 g/L) (Fig. 6f). In comparison, when strains were grown in minimal media containing glucose alone, glucose was also depleted by day 4 (Fig. 6a) and the biomass of all strains on this day peaked at an average of 6.8 ± 0.31 g/L (Fig. 6b). The statistically higher (*p* value < 0.01) biomass when grown with both glucose and xylose compared to the biomass accumulation in the presence of glucose alone indicates xylose utilization contributes to biomass accumulation for all the strains including wild type, however; as expected by the increased xylose usage, some strains were more efficient at using xylose for biomass than others. In both media, the biomass of all strains decreased after day 4; however, the biomass statistically decreased (*p* value < 0.01) more for strains grown in the presence of both glucose and xylose than glucose alone. As xylose was being consumed even as the biomass decreased, this suggests that xylose is not sufficient for biomass accumulation in this media.

### High copies of both endogenous T18 xylose isomerase and heterologous xylB xylulose kinase genes in T18 facilitates growth in rich media containing only xylose as carbon source

As glucose has been shown to repress xylose usage in other organisms, and xylose usage by the T18 transformants was still affected in the presence of glucose, we investigated whether providing the complete xylose isomerase and xylulose kinase pathway in T18 would allow the T18 strains to grow on xylose as the sole carbon source. Wild-type and transformants XI 16, XB, XI-XK 1, XI-XK 3, and XI-XK 7 were grown in either a defined minimal media or a rich media containing xylose as the sole carbon source. Minimal, if any, growth was observed for all the strains in minimal media containing xylose alone with little, if any, xylose used (data not shown), even though the cultures were inoculated at a 5× higher concentration than in the previous experiments.

**Fig. 7:**
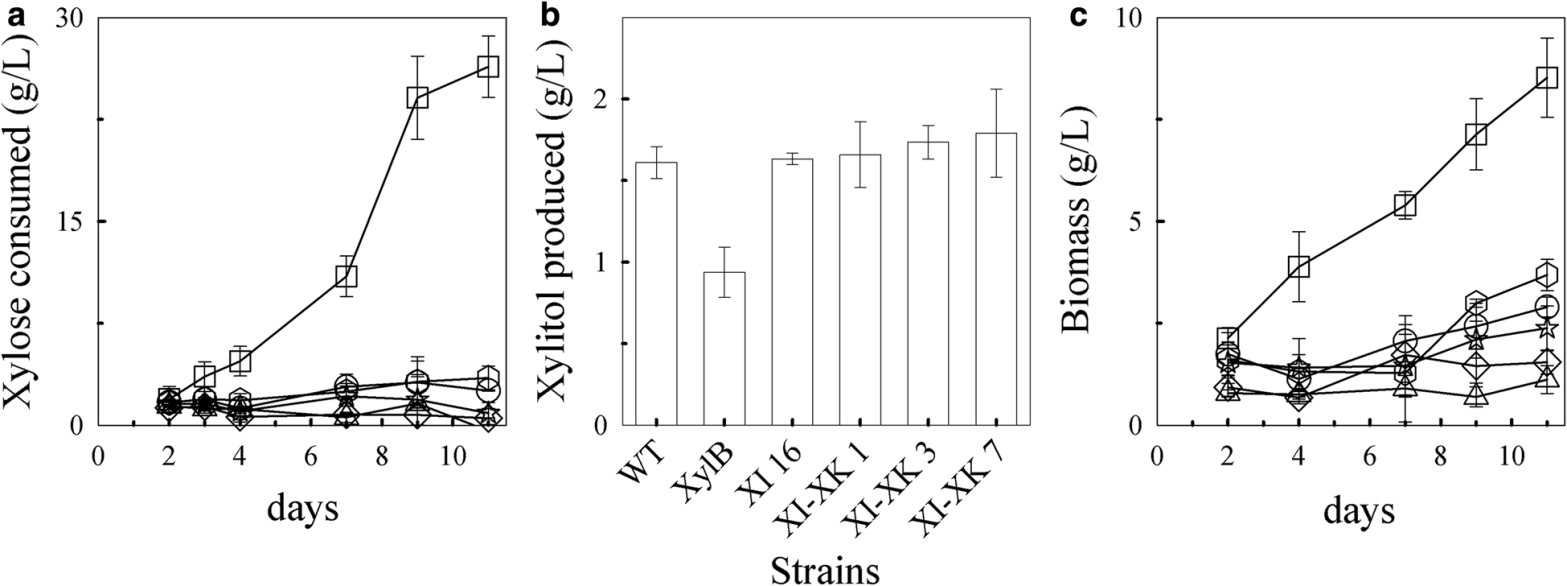
Growth of wild-type and transformants in rich media containing xylose as the main carbon source. **a** Xylose consumed, **b** xylitol produced, and **c** biomass made. Experiments were done in triplicate. The error bars represent standard deviation. Symbols: diamonds, WT; circles, XI 16; triangles, XB; hexagons, XI-XK 1; stars, XI-XK 3; squares, XI-XK 7

In rich media containing xylose as the main source of carbon, wild-type, transformants XI 16, XB, and XI-XK 1, and XI-XK 3 used very little, if any, xylose (Fig. 7a). In contrast, transformant XI-XK 7 was able to grow in this media using 24 g/L xylose and accumulating 8.5 g/L biomass (Fig. 7a, c). Despite consuming about 48% of the xylose present in the media, a negligible amount of xylitol (1.8 g/L) was produced by XI-XK 7 (Fig. 7b). The inability of the other strains to grow in this media indicates that not only are both genes required for growth in the presence of xylose alone but that a high copy number of both genes is needed. Furthermore, the inability of XI-XK 7, or any of the T18 strains, to grow in minimal media containing xylose as the sole carbon source points to additional nutrient(s) present in the rich media being required for xylose usage in the absence of other sugars. Presumably, as the strains can grow in minimal media containing both glucose and xylose, these are metabolites derived from pathways to which glucose contributes. Interestingly, unlike when grown in minimal media in the presence of glucose and xylose (Fig. 6f), the biomass of XI-XK 7 continued to increase over time further supporting that rich media contains key nutrients required for efficient xylose utilization.

### Fermentation of transformant with high copies of the xylose isomerase and xylulose kinase has enhanced xylose utilization, biomass, and lipid production

**Fig. 8:**
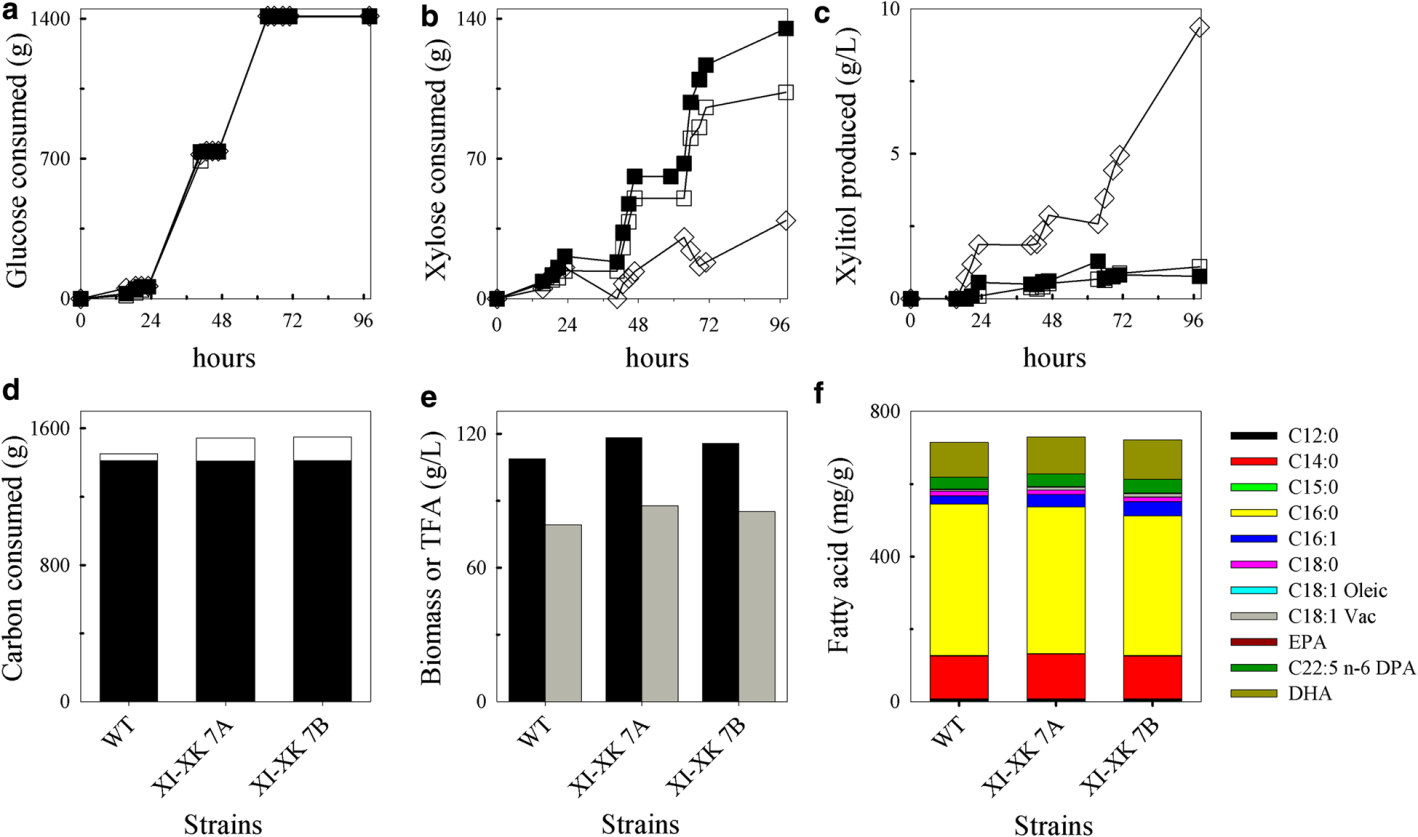
Fed-batch fermentations of wild-type and transformant XI-XK 7. **a** Glucose consumption, **b** xylose consumption, and **c** xylitol production throughout the fermentation. **d** Total carbon consumed at 96 h indicating the proportion of glucose (black portion) and xylose (white portion) used, **e** total biomass (black bar) and fatty acid (grey bar) produced, and **f** lipid profiles of transformant XI-XK 7 versus wild type (WT). Symbols: diamonds, WT; filled and unfilled squares, duplicate XI-XK 7 fermentations

The fermentation capability of transformant XI-XK 7, tested in duplicate, was compared to wild type in 7 L fermenters to determine whether the engineered strain was scalable for lipid production using xylose. As potential hemicellulosic feedstocks are expected to contain a combination of glucose and xylose, the cultures were batched in rich media containing glucose and xylose and fed equal amounts of glucose. The glucose consumption profiles were identical between wild-type T18 and the two XI-XK 7 fermentations (Fig. 8a), indicating that the XI-XK 7 strain does not have any significant growth delays compared to wild type under these conditions. To allow for xylose consumption, the strains were exposed to three equal rounds of glucose “starvation” over the course of 98 h. For both wild-type and XI-XK 7, xylose consumption occurred during these three glucose starvations periods; however, XI-XK 7 used much more xylose than wild type (Fig. 8b) and produced significantly less xylitol over the fermentation (Fig. 8c). By the end of the fermentation, wild type  consumed 1.45 kg of total carbon of which 39 g was xylose (Fig. 8d). In contrast, XI-XK 7 consumed an average of 1.55 kg total carbon and used 3.5-fold more xylose (average of 137 g) than wild-type. The efficient utilization of xylose by XI-XK 7 was characterized by an approximately tenfold reduction in xylitol in the supernatants (average of 0.94 g/L) compared to wild type (9.35 g/L) (Fig. 8c). The increased carbon used by XI-XK 7 resulted in about 1.1-fold more biomass (average of 117 g/L) than wild type (109 g/L) and 1.1-fold increase in total fatty acid (TFA) produced (87 g/L versus 79 g/L, respectively) (Fig. 8e). The increased xylose utilization by XI-XK 7 did not significantly impact the fatty acid profile of the strain relative to wild type, indicating that the genetic modifications did not impair or alter fatty acid production (Fig. 8f).

## Discussion

The cost of growing thraustochytrids for lipid production is highly dependent on the cost of the carbon feedstock. Keeping costs down relative to fossil fuel is especially important for the economical production of biofuel. Using hemicellulosic waste as a renewable carbon feedstock has been proposed [38]. As xylose makes up a significant portion of hemicellulose, efficient xylose utilization is required to maximize the cost-effectiveness of this stream.

As a highly effective oil producer, thraustochytrid T18 has commercial potential for biofuel production; however, its ability to grow in the presence of xylose has not been well studied. The association between thraustochytrids and decaying plant matter suggests that these organisms may use a variety of sugars including xylose. Indeed, we demonstrated that T18 is capable of consuming xylose but only when grown in the presence of glucose (Fig. 2). Diauxic growth was observed with T18 consuming xylose only after first depleting glucose. In addition, the growth of T18 in the presence of xylose resulted in a buildup of xylitol in the supernatant (Fig. 2b). This production of xylitol indicates the presence of a functional xylose reductase in T18 (Fig. 1). The presence of a xylulose kinase is also implied as not all of the xylose used was converted to and secreted as xylitol in wild-type T18, and all strains made more biomass in the presence of glucose and xylose compared to glucose alone. At this time, the presence of a xylitol dehydrogenase in T18 has not been experimentally confirmed, although putative genes with homology to known xylitol dehydrogenase have been identified. Similarly, although a putative xylulose kinase has also been identified, we have been unable to demonstrate any activity when this protein was expressed in vitro or when over-expressed in T18 (data not shown). The identity of genes encoding these proteins in T18 and their activity is being further investigated. It is unknown whether the xylitol present in the media is due to active secretion of xylitol or a result of cell lysis; however, xylitol accumulation may be due to co-factor imbalance as seen in other organisms [13]. Although T18 consumed xylose after glucose was depleted, the biomass decrease observed during this time was significantly sharper (Figs. 4 and 6) than the decrease seen following glucose depletion when the strains are grown in the presence of glucose alone (Figs. 4b and 6). It is possible this sharper decrease in biomass is due to the accumulation of extracellular xylitol, a bactericide [39, 40], resulting in further cell lysis when present in sufficient levels.

We also demonstrated that T18 encodes a functional and highly active xylose isomerase (Additional file 2: Figure S4). The presence of enzymes for two naturally occurring xylose metabolism pathways in a single organism was surprising and has not been previously reported, to our knowledge. The expression of this xylose isomerase gene in the wild type was high during xylose consumption and low when glucose was present suggesting some form of xylose metabolism regulation (Fig. 2c). At this time, it is not known whether the transcription of the xylose isomerase gene itself is repressed in the presence of glucose through catabolite repression or whether expression is directly linked to xylose transport into the cell which may itself be repressed in the presence of glucose. As the expression of *xi* and *xylB* are under the control of an α-tubulin promoter in the XI and XI-XK transformants, the repression of xylose metabolism gene expression by glucose should be alleviated, suggesting that xylose transport is most affected in these strains.

Over-expression of the xylose isomerase only resulted in improved xylose utilization when the gene was present at high copy numbers (Fig. 4). Despite increased xylose uptake, there was decreased xylitol production, indicating that there is a functional T18 xylulose kinase working in concert with the xylose isomerase. However, this xylulose kinase activity was not at sufficient levels to abolish xylitol production (Fig. 4) and biomass did not increase significantly over wild type in this xylose isomerase transformant (Fig. 4). Enhanced biomass production as well as xylose consumption required the over-expression of both the xylose isomerase and a xylulose kinase (Fig. 6d and f). Despite lower xylitol production, especially in transformant XI-XK 7, the biomass in minimal media still decreased when xylose was the only sugar being used, suggesting, perhaps, that xylose metabolism in the absence of a glucose metabolite results in a toxic byproduct. This is further supported by the inability of all strains to use xylose efficiently in minimal media containing xylose alone (data not shown). However, XI-XK 7 efficiently used xylose and produced significant biomass when grown in rich media containing xylose as a main carbon source (Fig. 7) and during glucose fed-batch fermentation (Fig. 8e). It is also possible that the complex nitrogen provided by the yeast extract and peptone in WDL media enhanced xylose utilization [41, 42]. Interestingly, the number of the xylose isomerase and xylulose kinase genes was critical for growth in xylose only rich media with approximately 26 xylose isomerase and 23 xylulose kinase copies being required for growth in this media (Fig. 7). A requirement for multiple xylose isomerase genes for enhanced xylose consumption has also been reported in S*. cerevisiae* [43], and optimal ratios of xylose reductase, xylitol dehydrogenase, and xylulose kinases have also been demonstrated [44, 45]. Although 2A cleavage occurs in T18, this cleavage is not 100% efficient (Additional file 2: Figure S6). There was a significant amount of the protein found as the un-cleaved Ble-2A-His-XI fusion and, presumably, Aph7-2A-XylB cleavage is not complete either. The functionality of the fusion proteins is unknown but if inactive, the limited cleavage of the proteins may contribute to a higher gene copy requirement for sufficient xylose isomerase and xylulose kinase activities to obtain enhanced xylose utilization. As the sequences flanking the 2A sequence in other organisms affect cleavage efficiency [46], additional 2A sequences are being tested in T18.

The lack of co-utilization of both glucose and xylose is a potential limit to efficient utilization of hemicellulosic waste streams by T18. Whether this inability to use both sugars simultaneously is due to catabolite repression or to a lack of transporters with higher xylose affinity is unknown. However, as T18 is able to uptake xylose, it encodes for sugar transporters that have some specificity to xylose. Identifying and potentially optimizing the specificity of these transporters for xylose may allow for co-utilization of glucose and xylose. Meanwhile, modifying the fermentation conditions allowed a significant amount of biomass and oil to be produced in the presence of both glucose and xylose with both sugars being used and little xylitol buildup in the media (Fig. 8e). Alternatively, the ability of the engineered strain to grow in media containing xylose alone makes it attractive for growth on waste streams with higher proportions of xylose than glucose such as corn stover or birch wood [47–49]. In addition, using xylose may be advantageous for lipid production, as xylose may increase acetyl-CoA production [50, 51].

Homologous recombination of exogenous linear DNA was found to be very efficient in T18. Upon transformation,  the linear DNA constructs, flanked by approximately 1 kb regions of homology corresponding to the α-tubulin promoter and terminator, integrated into the targeted α-tubulin locus and replaced the α-tubulin gene in many transformants. Transformation of T18 with linear DNA sometimes resulted in strains with concatemers, as previously observed in *Chlamydomonas reinhardtii* and *Physcomitrella patens* [52, 53]. The mechanism for concatemer formation in T18 is unclear; however, multiple insertion and/or rearrangement of transgenes following particle bombardment has been also observed in plants [54–56]. The mechanism for the doubling or reduction of the xylose isomerase genes following transformation of transformant XI 16 is unknown. It is possible that the reduction in *ble*-*2A*-*his*-*xi* copy number in transformant XI-XK 3 was caused by homologous recombination of the transformed *aph7*-*2A*-*xylB* cassette replacing integrated copies of the *ble*-*2A*-*his*-*xi* cassette as they are both flanked by the same ~ 1 kb homology arms. Nonetheless, the resulting multiple gene copies were found to be critical for xylose consumption; as such, to better understand and possibly take advantage of this phenomenon, the genomes of these strains will be further analyzed to determine how the DNA constructs were integrated.

## Conclusions

In conclusion, we demonstrate that thraustochytrid T18 encodes for the first two enzymes of two native xylose metabolism pathways. Over-expression of an endogenous xylose isomerase and a heterologous xylulose kinase in T18 resulted in strains consuming significant amounts of xylose to make biomass and lipid. As some thraustochytrid strains, including T18, are already being used at industry scale, this work demonstrates the potential of using these microorganisms for commercial lipid production from hemicellulosic waste streams.

## Additional files

**Additional file 1: Table S1.** Plasmids used in this study. **Table S2.** Primers used for cloning. **Table S3.** Primers used for qPCR.

**Additional file 2: Figure S1.** Glucose (Glc) and xylose (Xyl) consumption by wild-type over time and xylitol (Xyt) identification. A) Chromatograms of the culture supernatant on days 0 (dashed red line), 1, 2, 3 (solid black line), 4 (dashed cyan line), and 7 (solid blue line). B) Chromatograms of the culture supernatant from day 7 before (solid blue line) and after (dashed line) spiking with xylitol. C) Mass spectrometry analysis of the unknown peak. **Figure S2.** SDS-PAGE of metal chelation fractions used for *in vitro* protein activity assays. Lane 1: molecular weight marker, lane 2: fraction containing histidine-tagged putative xylose isomerase, lane 3: fraction containing mostly the co-eluting band. **Figure S3.** Effect of temperature on (A) XylA and (B) T18 XI activity on xylose and xylulose. The mean is plotted with the error bars representing the highest and lowest values of duplicate assays. Symbols: diamonds, xylose; squares, xylulose. **Figure S4.** Dose dependence of (A) XylA and (B) T18 XI with xylose (diamonds) or xylulose (squares). A negative control with fraction containing the 39 kDa co-eluted protein but undetectable levels of 52 kDa protein is also shown (filled symbols). XylA assays were done in duplicate at 30°C; the mean is plotted with the error bars representing the highest and lowest values. T18 xylose isomerase assays were done in triplicate at 50°C, the mean is plotted, and the error bars represent the standard deviation. **Figure S5.** Southern blot analysis of the wild-type (WT) and xylose isomerase (XI) transformants. Blots were probed with sequences from A) an α-tubulin-locus area and B) *ble*. Restriction maps of C) the intact wild-type α-tubulin locus and D) the same locus after homologous recombination replacing the α-tubulin ORF with *ble-2A-his-xi*. The location of the *ble* and α-tubulin loci probes are indicated as black rectangles. HR represents the approximately 1kb homology arms available for homologous recombination corresponding to the α-tubulin promoter and terminator regions flanking the *ble-2A-his-xi* construct. Genomic DNA was digested with HindIII and ScaI. A 2.9 kb band is expected for wild type with the α-tubulin area probe. Based on this hypothetical knockout transformant map, a 5.7 kb band is expected with both probes. The molecular weight markers (MW) and corresponding sizes are indicated. **Figure S6.** Western blot analysis of cell extracts from wild-type T18 (WT) and XI transformants. Blots were probed with A) anti-2A antibodies and B) anti-his antibodies. Protein extracts were incubated for 30 min at 37°C or 5 min at 100°C prior separation by SDS-PAGE. Arrows indicate the bands representing Ble-2A-His-XI (1), His-XI (2), and Ble-2A (3). **Figure S7.** *In vitro* activity assays with cell extracts from wild-type and XI transformants *In vitro* activity assays with cell extracts from wild-type and XI transformants. Experiments were done in triplicate with A) xylose or B) xylulose at 50°C. Error bars represent standard deviation. Symbols: diamonds, wild-type; squares, XI 4; triangles, XI 6; hexagons, XI 8; and circles, XI 16. **Figure S8.** Southern blot analysis of XI-XK transformants. Blots were probed with sequences specific to A) an α-tubulin area, B) *ble*, and C) *aph7*. D) The restriction map of the pJB47 plasmid encoding the α-tubulin promoter-*aph7-2A*-*xylB*-α-tubulin terminator construct used to transform the parental strain XI 16. The location of *aph7* probe is indicated as a black bar. HR represents the homology arms available for homologous recombination. Genomic DNA was digested with ScaI-SbfI or ScaI-HindIII. The order of the samples is the same on all blots. The expected band sizes for the WT α-tubulin loci detected by the α-tubulin probe are 7.8 kb and 2.9 kb for the ScaI-SbfI and ScaI-HindIII digests, respectively. The molecular weight markers (MW) and corresponding sizes are shown. **Figure S9.** Southern blot and qPCR analyses of Wild-type (WT), XI 8 and XB transformants. Blots were probed with sequences from A) an α-tubulin-locus area and B) *ble*. C) Restriction map of the α-tubulin locus after homologous recombination replacing the α-tubulin ORF with *ble-2A-xylB*. The location of the *ble* and α-tubulin loci probes is indicated as black rectangles. HR represents the approximately 1kb homology arms available for homologous recombination corresponding to the α-tubulin promoter and terminator regions flanking the *ble-2A-xylB* construct. Genomic DNA was digested with StuI or NotI. Based on this hypothetical knockout XB transformant map, the NotI digest should result in a 2.8 kb band with the α-tubulin probe and the StuI digest should result in a 2.8 kb band with the *ble* probe. The molecular weight markers (MW) and corresponding sizes are indicated. D) The gene copy number of the transgenic *ble* gene was measured by qPCR. Error bars represent the higher and lower relative quantity limits. **Figure S10.** Xylose isomerase and xylulose kinase activities in wild-type and XI-XK transformants. Experiments were done in triplicate with A) xylose or B) xylulose. Xylose isomerase C) and xylulose kinase D) activities were calculated from the xylulose reactions. The error bars represent standard deviations. Symbols: diamonds, WT; circles, XI 16; triangles, XB; hexagons, XI-XK 1; stars, XI-XK 3; squares, XI-XK 7.

## Abbreviations

DHA: docosahexaenoic acid
EPA: eicosapentaenoic acid
HPLC: high-performance liquid chromatography
REU: relative expression units
RI: refractive index
RIU: refractive index units
TFA: total fatty acids
XI: xylose isomerase
XR: xylose reductase
XD: xylitol dehydrogenase
XK: xylulose kinase
WDL: Windust light
