# Supplementary material for: Engineering xylose metabolism in thraustochytrid T18
Source: Biotechnol Biofuels. 2018 Sep 17;11:248. doi: 10.1186/s13068-018-1246-1 (PMC6139898; doi:10.1186/s13068-018-1246-1)
Supplement: Supplementary file 1 — Additional file 1: Table S1. Plasmids used in this study. Table S2. Primers used for cloning. Table S3. Primers used for qPCR. [file 13068_2018_1246_MOESM1_ESM.pdf]

Additional file 1

Additional file 1: Table S1: Plasmids used in this study

Plasmids	Relevant characteristics	References
pCR2.1-Topo	AmpR. *E. coli* cloning vector	Invitrogen
pTopoXyloIso	AmpR. T18 xylose isomerase in pCR2.1-Topo	This study
pET23-MTF	AmpR. pET23 derivative containing N-terminal histidine tag and T7 promoter	[1]
pET23-isohis	AmpR. His-tagged T18 xylose isomerase in pET23-MTF	This study
pYES2	AmpR. Yeast expression plasmid with GAL1 promoter and *URA3*.	Invitrogen
pYES2-isohis	AmpR. Inducible His-tagged T18 xylose isomerase for protein expression in yeast	This study
pTopoXylA	AmpR. W3110 *xylA* in pCR2.1-Topo	This study
pET23-XylAhis	AmpR. His-tagged *E. coli W3110 xylA* for expression in *E. coli* under a T7 promoter	This study
pJB5	KanR. pUC5, α-tubulin promoter, *ble-2A*, α-tubulin terminator	This study
pα-tub isohis	KanR. His-tagged T18 xylose isomerase cloned downstream of *ble-2A* of pJB5. Zeocin selection for T18	This study

pChlamy_3	AmpR. Source of *aph7* for hygromycin selection in T18	Invitrogen
pJB47	KanR. T18 codon optimized *E. coli W3110* x*ylB* under control of α-tubulin promoter and terminator. *aph7-2A* for hygromycin selection in T18	This study

Additional file 1: Table S2: Primers used for cloning

Primer	Sequence[a]	Restriction site
AMJP1	ATA TCT AGA **ATG** GAG TTC TTC CCC GAG	XbaI
AMJP2	TCC CCC GGG **TTA** GGA AAT GTA GTG GTT GAG	SmaI
AMJP11	ATA TCT AGA **ATG** CAA GCC TAT TTT GAC CAG	XbaI
AMJP12	CGC GGA TCC **TTA** TTT GTC GAA CAG ATA ATG GT	BamHI
AMJP15	CAC AC**A TG**T CGA TGG AGT TCT TCC CCG AG	AflIII
AMJP16	GA AGA TCT **TTA** GGA AAT GTA GTG GTT GAG	BglII
AMJP20	AAT ACC **ATG** GGC AT GCA AGC CTA TTT TGA CCA G	NcoI
AMJP39	GAT ATG CAT A**AT G**CA CCA CCA CCA CCA CCA	NsiI
AMJP40	CAT GCG GCC GC**T TA**G GAA ATG TAG TGG TTG AG	NotI
JBo28	ATT ATT TCT AGA ATG ACA CAA GAA TCC CTG TTA C	XbaI
JBo29	ATT ATT GGT ACC GGC GCC GGG GGC GGT G	KpnI

[a]. The restriction sites are underlined and the start or stop codons are in bold.

Additional file 1: Table S3: Primers used for qPCR

Gene	Primer name	Sequence
actin	actin2fwd	CGT CCT GCG CAT TGA TCT TG
	actin2rvs	GGC GAG CTT CTC CTT GAT GT
GAPDH	GAPDH2fwd	GGC GTC AAC CAC AAG GAG TA
	GAPDH2rvs	TGT CGT TGA TGA CCT TGG CA
xi	isoqrtpcrfwd1	TGA GAT TGG GGA AAC CTC GC
	isoqrtpcrrvs1	TCT TGA CTG GTT CCG TGT CG
ble	ble2HR-F	GAC GAC GTG ACC CTG TTC AT
	ble2HR-R	TCC CGG AAG TTC GTG GAC A
aph7	HRo37	CAG CGT GCT TGC AGA TTT GA
	HRo38	TGC TTG AGA CAG CGA CAG AG

Additional Reference

1. Newton DT, Mangroo D. Mapping the active site of the Haemophilus influenzae methionyl-tRNA formyltransferase: residues important for catalysis and tRNA binding. Biochem. J. 1999;339:63–9.
