# Supplementary material for: Engineering xylose metabolism in thraustochytrid T18
Source: Biotechnol Biofuels. 2018 Sep 17;11:248. doi: 10.1186/s13068-018-1246-1 (PMC6139898; doi:10.1186/s13068-018-1246-1)
Supplement: Supplementary file 2 — Additional file 2: Figure S1. Glucose (Glc) and xylose (Xyl) consumption by wild-type over time and xylitol (Xyt) identification. A) Chromatograms of the culture supernatant on days 0 (dashed red line), 1, 2, 3 (solid black line), 4 (dashed cyan line), and 7 (solid blue line). B) Chromatograms of the culture supernatant from day 7 before (solid blue line) and after (dashed line) spiking with xylitol. C) Mass spectrometry analysis of the unknown peak. Figure S2. SDS-PAGE of metal chelation fractions used for in vitro protein activity assays. Lane 1: molecular weight marker, lane 2: fraction containing histidine-tagged putative xylose isomerase, lane 3: fraction containing mostly the co-eluting band. Figure S3. Effect of temperature on (A) XylA and (B) T18 XI activity on xylose and xylulose. The mean is plotted with the error bars representing the highest and lowest values of duplicate assays. Symbols: diamonds, xylose; squares, xylulose. Figure S4. Dose dependence of (A) XylA and (B) T18 XI with xylose (diamonds) or xylulose (squares). A negative control with fraction containing the 39 kDa co-eluted protein but undetectable levels of 52 kDa protein is also shown (filled symbols). XylA assays were done in duplicate at 30°C; the mean is plotted with the error bars representing the highest and lowest values. T18 xylose isomerase assays were done in triplicate at 50°C, the mean is plotted, and the error bars represent the standard deviation. Figure S5. Southern blot analysis of the wild-type (WT) and xylose isomerase (XI) transformants. Blots were probed with sequences from A) an α-tubulin-locus area and B) ble. Restriction maps of C) the intact wild-type α-tubulin locus and D) the same locus after homologous recombination replacing the α-tubulin ORF with ble-2A-his-xi. The location of the ble and α-tubulin loci probes are indicated as black rectangles. HR represents the approximately 1kb homology arms available for homologous recombination corresponding to the α-tubulin [file 13068_2018_1246_MOESM2_ESM.pdf]

Additional file 2

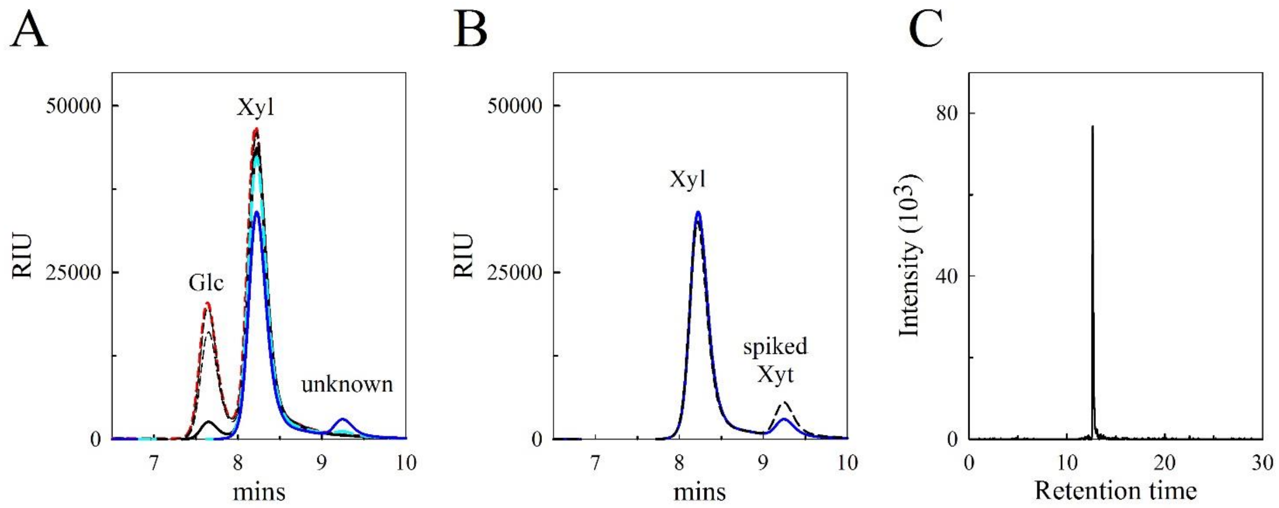

Additional file 2: Figure S1: Glucose (Glc) and xylose (Xyl) consumption by wild-type over time and xylitol (Xyt) identification. A) Chromatograms of the culture supernatant on days 0 (dashed red line), 1, 2, 3 (solid black line), 4 (dashed cyan line), and 7 (solid blue line). B) Chromatograms of the culture supernatant from day 7 before (solid blue line) and after (dashed line) spiking with xylitol. C) Mass spectrometry analysis of the unknown peak.

When wild-type was grown in the presence of glucose and xylose, glucose was the primary sugar used on day 1 and day 2 as indicated by the reduction of the glucose peak over this period. Only when glucose levels were low (day 3) did the concentration of xylose in the medium start decreasing, with most of the decrease occuring between day 4 and 7. By day 7 (Additional file 2: Figure S1A, blue line) an additional peak migrating at 9.2 minutes was detected. Compared to pure standards, this peak migrated at the same time as xylitol and, indeed, spiking the supertantant from day 7 with a small amount of xylitol resulted in an increase of this peak (Additional file 2: Figure S1B). The unknown peak was purified and analyzed by mass spectrometry by an external laboratory confirming it to be xylitol (Additional file 2: Figure S1C).

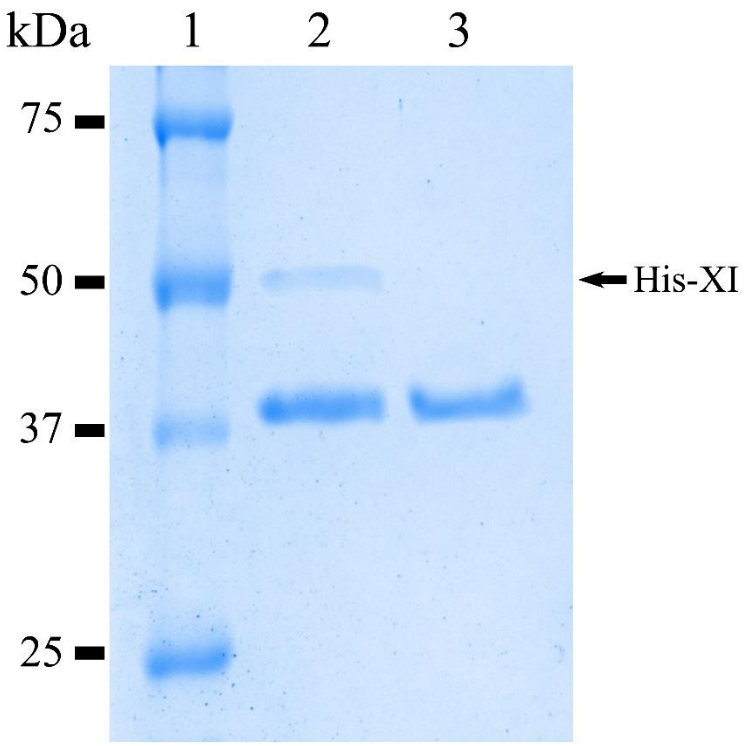

Additional file 2: Figure S2: SDS-PAGE of metal chelation fractions used for *in vitro* protein activity assays. Lane 1: molecular weight marker, lane 2: fraction containing histidine-tagged putative xylose isomerase, lane 3: fraction containing mostly the co-eluting band.

The gene encoding for the putative T18 xylose isomerase was cloned from T18 with an N-terminal histidine tag under a *GAL1* promoter to allow purification of the protein (with a calculated molecular weight of 50.8 kDa with the tag) by metal chelation after over-expression in yeast. Despite optimizing the imidazole concentrations for elution, fractions containing the putative T18 xylose isomerase (migrating at approximately 52 kDa) also contained a prominent co-eluting ~39 kDa protein (Additional file 2: Figure S2, lane 3). Based on band intensity, the T18 protein is calculated to be 13% of the fraction.

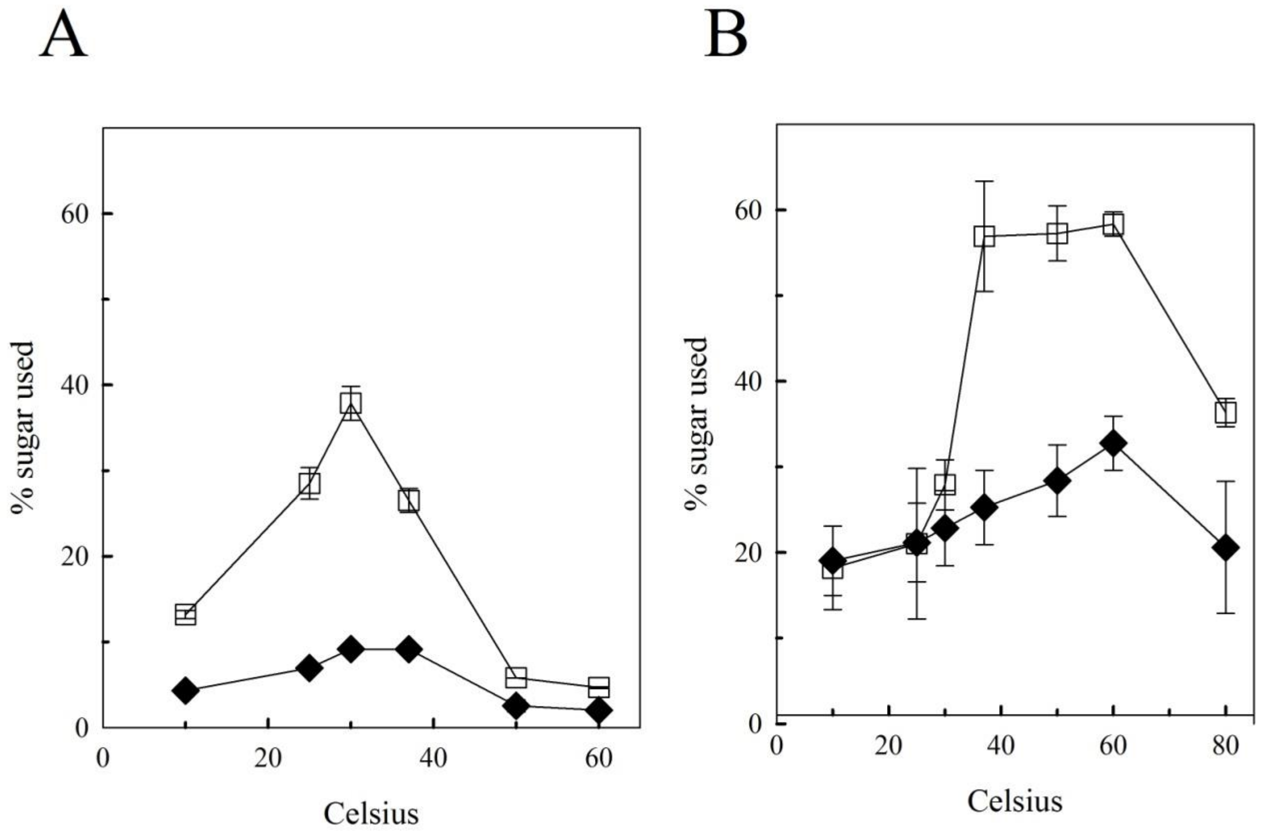

Additional file 2: Figure S3: Effect of temperature on (A) XylA and (B) T18 XI activity on xylose and xylulose. The mean is plotted with the error bars representing the highest and lowest values of duplicate assays. Symbols: diamonds, xylose; squares, xylulose.

To determine whether the T18 enzyme followed similar kinetics as XylA, the effect of temperature on the activity of the enzyme was determined using both sugars. Whereas XylA activity peaked at 30°C with 9% of xylose and 33% of xylulose interconverted (Additional file 2: Figure S3A), the activity of T18 xylose isomerase increased significantly at 37°C and remained high even at 60°C with approximately 30% of xylose and 57% of xylulose interconverted (Additional file 2: Figure S3B). Further experiments were done at 50°C to increase activity on xylose.

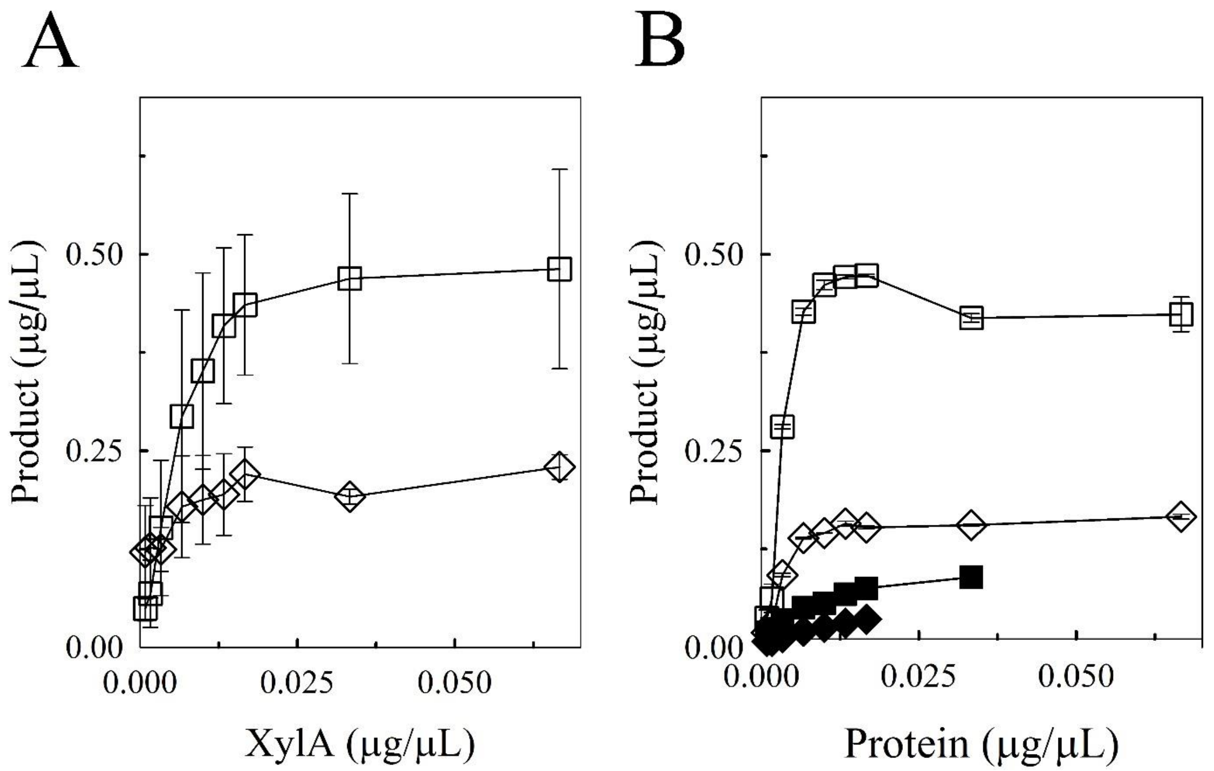

Additional file 2: Figure S4: Dose dependency of (A) XylA and (B) T18 XI with xylose (diamonds) or xylulose (squares). A negative control with fraction containing the 39 kDa co-eluted protein but undetectable levels of 52 kDa protein is also shown (filled symbols). XylA assays were done in duplicate at 30°C, the mean is plotted with the error bars representing the highest and lowest values. T18 xylose isomerase assays were done in triplicate at 50°C, the mean is plotted and the error bars represent the standard deviation.

Due the inherent increased isomerase activity on xylulose compared to xylose at equilibrium, the analysis on xylulose is described in detailed; however, a similar trend was observed with xylose. XylA activity on xylulose peaked at 0.013 µg/µL protein with about 0.41 µg/µL xylose made (Additional file 2: Figure S4A) whereas the same amount of total protein containing T18 xylose isomerase made 0.47 µg/µL xylose (Additional file 2: Figure S4B). In contrast, only 0.07 µg/µL xylose was made when xylulose was incubated with 0.013 µg/µL of total protein containing the 39 kDa protein and undetected amounts of T18 xylose isomerase,

confirming that the 52 kDa protein is responsible for the xylose isomerase activity. As the xylose isomerase made up 13% of the total protein, 0.013 μg/μL of total protein would represent 0.0017 μg/μL of T18 xylose isomerase demonstrating that T18 xylose isomerase has a much higher isomerase activity than XylA in these assays.

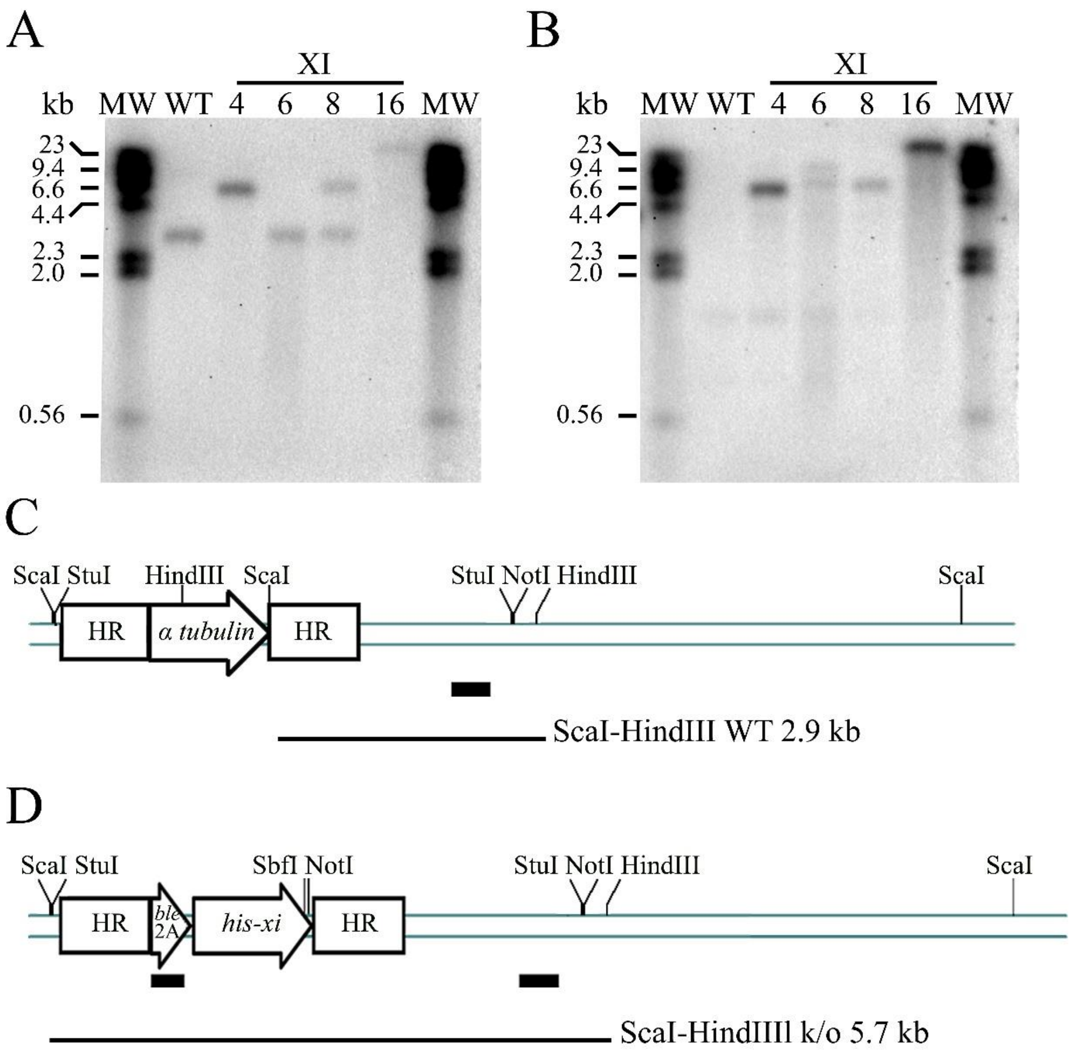

Additional file 2: Figure S5: Southern blot analysis of the wild-type (WT) and xylose isomerase (XI) transformants. Blots were probed with sequences from A) an α-tubulin-locus area and B) *ble*. Restriction maps of C) the intact wild-type α-tubulin locus and D) the same locus after homologous recombination replacing the α-tubulin ORF with *ble-2A-his-xi*. The location of the *ble* and α-tubulin loci probes are indicated as black rectangles. HR represents the approximately 1kb homology arms available for homologous recombination corresponding to the α-tubulin promoter and terminator regions flanking the *ble-2A-his-xi* construct. Genomic DNA was digested with HindIII and ScaI. A 2.9 kb band is expected for wild-type with the α-tubulin area probe. Based on this hypothetical knockout transformant map, a 5.7 kb band is expected with both probes. The molecular weight markers (MW) and corresponding sizes are indicated.

Four representative transformants (XI 4, XI 6, XI 8, and XI 16) were examined for homologous recombination at an α-tubulin locus by Southern blotting using an α-tubulin-locus-specific probe (Additional file 2: Figure S5A) and a *ble*-specific probe (Additional file 2: Figure S5B). The exact expected size of the fragment for a knocked out α-tubulin gene is unknown due to gaps in our knowledge regarding the mechanisms of homologous recombination in thraustochytrids. Nonetheless, assuming a knockout strain would involve the full replacement of this particular α-tubulin gene (Additional file 2: Figure S5C) by the *ble-2A-his-xi* construct between the homology arms, the expected map for a knockout is shown (Additional file 2: Figure S5D). Based on the Southern blot analysis and evidence that some thraustochytrids are diploids, XI 6 was determined to have the transgenes randomly inserted in its genome, XI 8 was determined to be a heterozygote, with the transgene replacing one of the two copies of the α-tubulin at the targeted locus, and XI 4 and XI 16 were concluded to be homozygotes. Interestingly, in XI 16 the much larger (~23 kb) band at the α-tubulin locus indicates the presence of a construct larger than a single copy of the *ble-2A-his-xi* construct seen in XI 4 and XI 8.

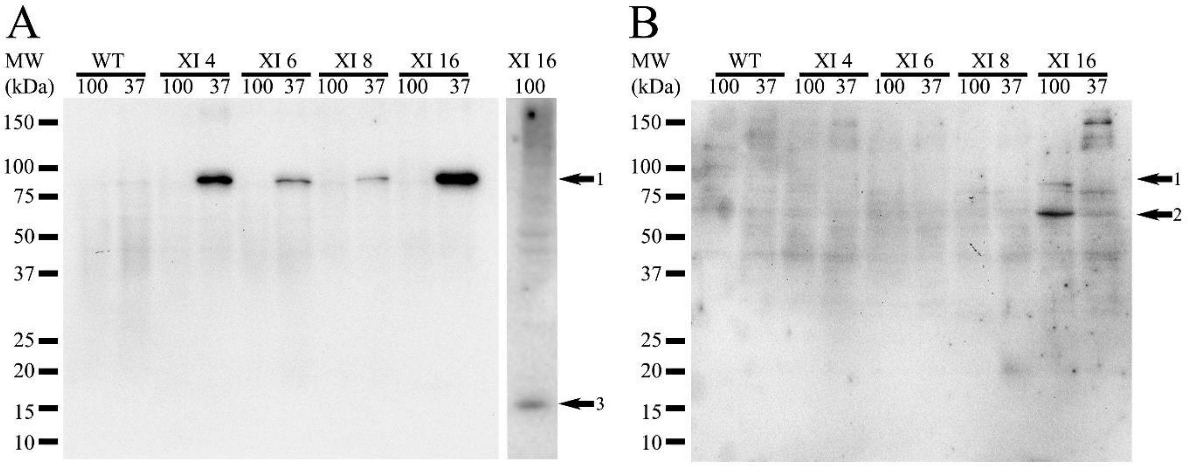

Additional file 2: Figure S6: **Western blot analysis of cell extracts from wild-type T18 (WT) and XI transformants.** Blots were probed with A) anti-2A antibodies and B) anti-his antibodies.

Protein extracts were incubated for 30 minutes at 37°C or 5 minutes at 100°C prior separation by SDS-PAGE. Arrows indicate the bands representing Ble-2A-His-XI (1), His-XI (2), and Ble-2A (3).

2A sequences are often used to introduce multiple proteins on a single transcript whereby cleavage occurs through ribosome "skipping" at 2A site. Although 2A cleavage occurs in most organisms, it has not been documented in thraustochytrids. The expression of the Ble-2A-His-XI fusion protein as well as the expected cleavage products were determined by Western blot hybridization using anti-histidine tag and anti-2A antibodies. In all four XI transformants, the Ble-2A-His-XI fusion (expected 64 kDa) was detected at relative intensities which correlated with their *xi* gene copies (Additional file 2: Figure S6A). The cleaved Ble-2A (expected 14 kDa) and His-tagged XI proteins (expected 50 kDa); however, were only detected in transformant XI 16 (Additional file 2: Figure S6A and B). The increased observed molecular weights of the His-XI protein and Ble-2A-His-Xi fusion protein suggest potential post-translational modifications in this eukaryotic organism as has been observed in other organisms [1,2]. The absence of the cleaved bands in the other XI transformants indicates that 2A cleavage is not efficient in T18 however, the detection of all three protein species in transformant XI 16 confirms some level of 2A cleavage does occur in T18. Furthermore, the observed increased isomerase activity in the XI strains relative to the wild-type indicates that the fusion protein has activity and/or there is sufficient cleavage to observe some isomerase activity within the strains.

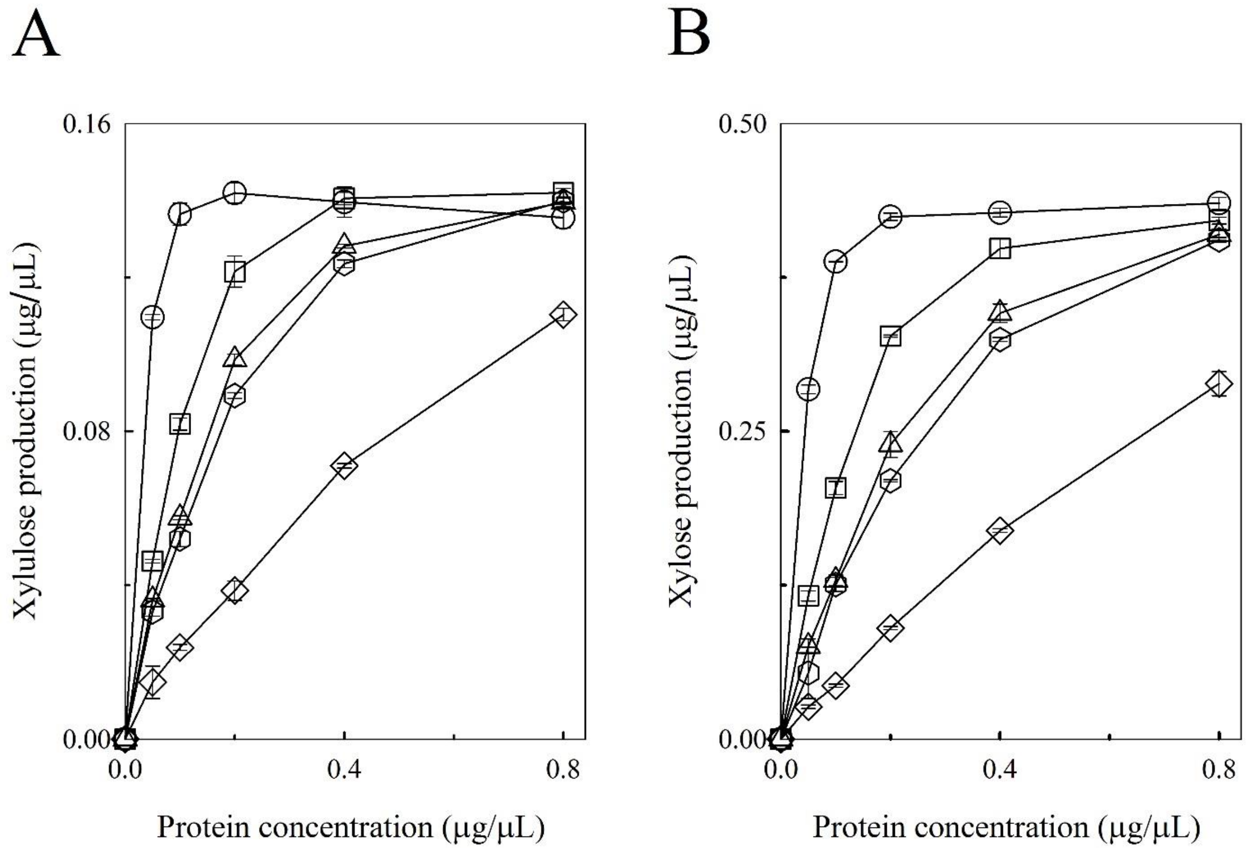

Additional file 2: Figure S7: *In vitro* activity assays with cell extracts from wild-type and XI transformants. Experiments were done in triplicate with A) xylose or B) xylulose at 50°C. Error bars represent standard deviation. Symbols: diamonds, wild-type; squares, XI 4; triangles, XI 6; hexagons, XI 8; and circles, XI 16.

Protein dose dependency was observed for the four transformant strains demonstrating that the histidine-tagged xylose isomerase is functional *in vitro*. Comparing xylose isomerase activities at the same total protein concentration revealed that the increased copy number of the His-tagged xylose isomerase gene in the transformants correlated with a corresponding increase in xylose isomerase activity (Additional file 2: Figure S7A and B, Table 2). Protein extracts from XI 8, with its extra xylose isomerase copy, had 2.3x higher isomerase activity on both xylose and xylulose compared to protein extracts from wild-type at 0.2 μg/μL. Protein extracts from XI 6, which encodes for two additional xylose isomerase copies, had 2.7x times more activity on both

sugars compared to protein extracts from wild-type at 0.2 μg/μL but only a slight increase over XI 8 at the same protein concentration. In turn, protein extracts from XI 4, with its four additional xylose isomerase copies, converted 3.1x and 3.6x more of both than protein extracts from wild-type and 1.2x and 1.4x more than protein extracts from XI 6 at this protein concentration. Notably, protein extracts from XI 16, with the highest xylose isomerase copies, converted 3.7 to 4.7x more of both sugars than protein extracts from wild-type.

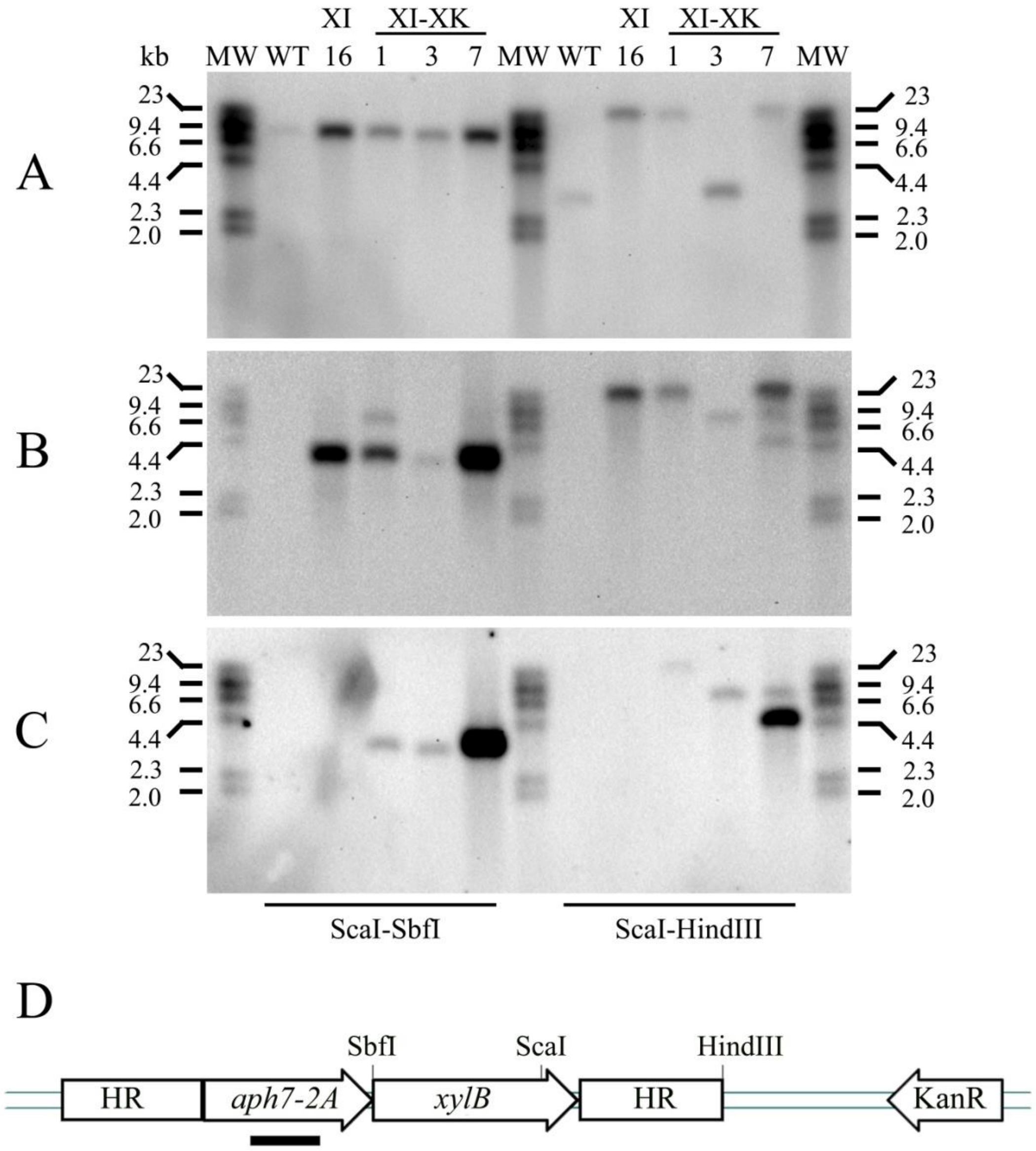

Additional file 2: Figure S8: Southern blot analysis of XI-XK transformants. Blots were probed with sequences specific to A) an α-tubulin area, B) *ble*, and C) *aph7*. D) The restriction map of the pJB47 plasmid encoding the α-tubulin promoter-*aph7-2A-xylB*-α-tubulin terminator construct used to transform the parental strain XI 16. The location of *aph7* probe is indicated as a black bar. HR represents the homology arms available for homologous recombination. Genomic DNA was digested with ScaI-SbfI or ScaI-HindIII. The order of the samples is the same on all blots. The expected band sizes for the WT α-tubulin loci detected by the α-tubulin probe are 7.8

kb and 2.9 kb for the ScaI-SbfI and ScaI-HindIII digests respectively. The molecular weight markers (MW) and corresponding sizes are shown.

As the *xylB* construct shares the α-tubulin promoter and terminator homology arms in the XI 16 parental strain, the potential replacement of the *ble-2A-his-xi* construct by the *aph7-2A-xylB* construct was examined using probes specific for the α-tubulin locus, *ble*, and *aph7*. Changes in banding patterns with both the α-tubulin locus and *ble* probes were observed in all three strains compared to the parental strain in two separate combinations of restriction enzymes (ScaI-SbfI and ScaI-HindIII). As the structure of the multiple *ble-2A-his-xi* concatemer construct in the parental strain XI 16 is unknown, the expected band sizes for each digest could not be calculated. Nonetheless, the changes in banding pattern are consistent with the insertion of *aph7-2A-xylB* within the parental XI 1 *ble-2A-his-xi* concatemer.

Specifically, a smaller band in XI-XK 3 was observed in the ScaI-HindIII digest following hybridization with the α-tubulin-locus specific probe (3.3 kb band in Additional file 2: Figure S8A) and the *ble*-specific probe (8.2 kb band in Additional file 2: Figure S8B) compared to the parental strain suggesting a loss of *ble-2A-his-xi* gene copies. The presence of just one band with both probes also suggests that the same rearrangement occurred on both chromosomes of XI-XK 3. In addition, the 8.2 kb ScaI-HindIII band was detected in this transformant with the *aph7* specific probe indicating that this fragment also contained the *aph7-2A-xylB* transgene (Additional file 2: Figure S8C). Rearrangement of *ble-2A-his-xi* was also observed in XI-XK 1 as two ScaI-SbfI *ble*-specific bands (7.2 kb and 3.9 kb) were detected (Additional file 2: Figure S8B) compared to the one band in the parent. The 3.9 kb band also hybridized with the *aph7* probe (Additional file 2: Figure S8C) confirming that the *ble-2A-his-xi* rearrangement was due to the insertion of the *aph7-2A-xylB* transgenes. Finally, rearrangement of *ble-2A-his-xi* was observed in XI-XK 7 with the presence of multiple *ble*-specific bands (Additional file 2: Figure

S8B), two of which (8.8 kb and 4.6 kb) also reacted with the *aph7* probe (Additional file 2: Figure S8C).

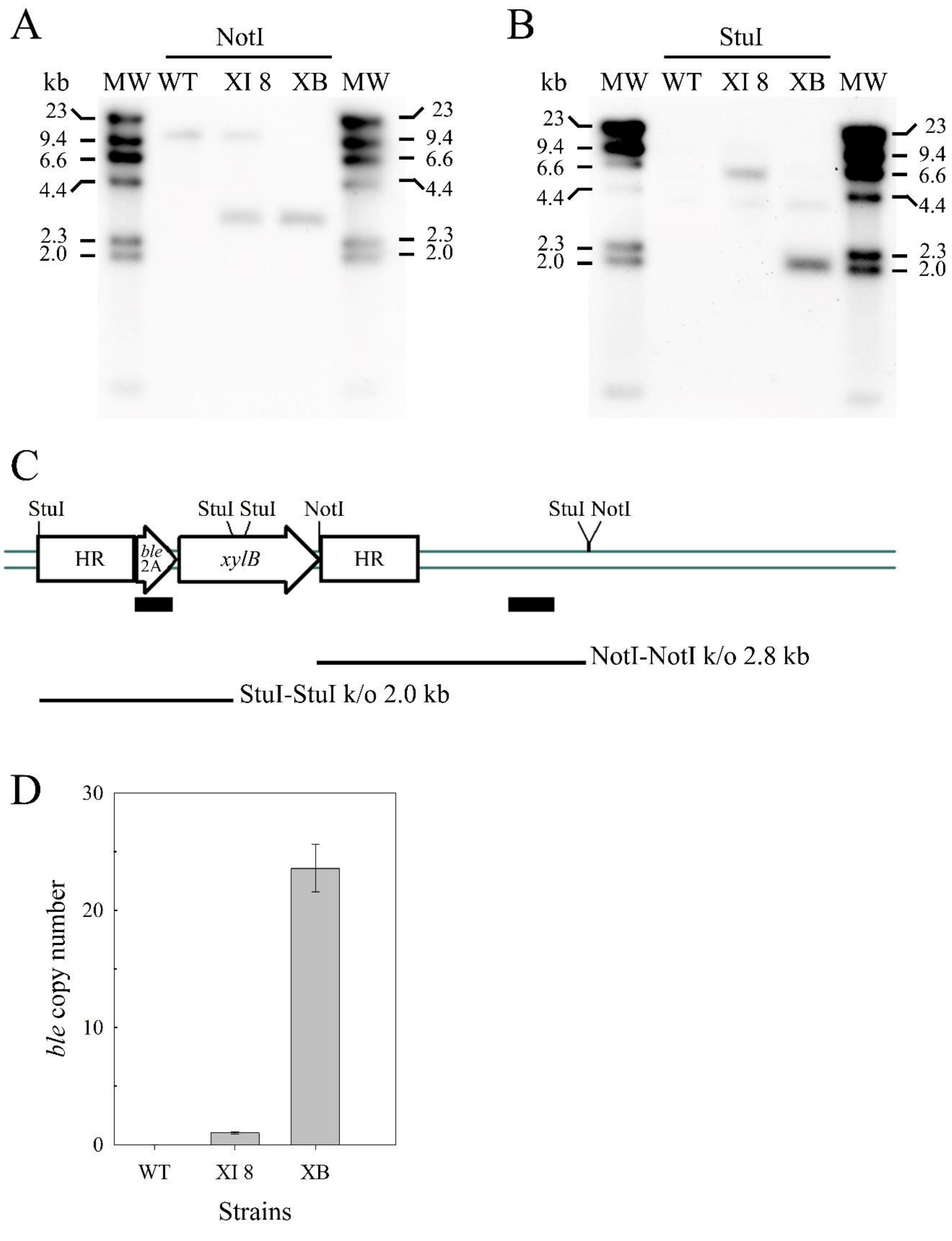

Additional file 2: Figure S9: Southern blot and qPCR analyses of Wild-type (WT), XI 8 and XB transformants. Blots were probed with sequences from A) an α-tubulin-locus area and B) *ble*. C) Restriction map of the α-tubulin locus after homologous recombination replacing the α-tubulin ORF with *ble-2A-xylB*. The location of the *ble* and α-tubulin loci probes are indicated

as black rectangles. HR represents the approximately 1kb homology arms available for homologous recombination corresponding to the α-tubulin promoter and terminator regions flanking the *ble-2A-xylB* construct. Genomic DNA was digested with StuI or NotI. Based on this hypothetical knockout XB transformant map, the NotI digest should result in a 2.8 kb band with the α-tubulin probe and the StuI digest should result in a 2.0 kb band with the *ble* probe. The molecular weight markers (MW) and corresponding sizes are indicated. D) The gene copy number of the transgenic *ble* gene was measured by qPCR. Error bars represent the higher and lower relative quantity limits.

Of the several *ble-2A-xyl* transformants obtained, the XB transformant was chosen predominantly for its high xylulose kinase activity in cell extracts assays (Additional file 2: Figure S10). Probing for the α-tubulin area and *ble* revealed that this transformant was a homozygote knockout of the α-tubulin gene (Additional file 2: Figure S9A and B). Probing for the α-tubulin area of NotI digested wild-type is expected to result in a 5.1 kb band. With the α-tubulin area probe, the NotI digested heterozygote XI 8 is expected to result in the same wild-type 5.1 kb band as well as the same 2.8 kb band as XB. With the StuI digest of XI 8 and *ble* probe a 5.6 kb band is expected. All expected bands were observed with both probes and digests.

Finally, qPCR analysis on the transformant indicated the presence of up to 23 copies of *ble-2A-xylB*.

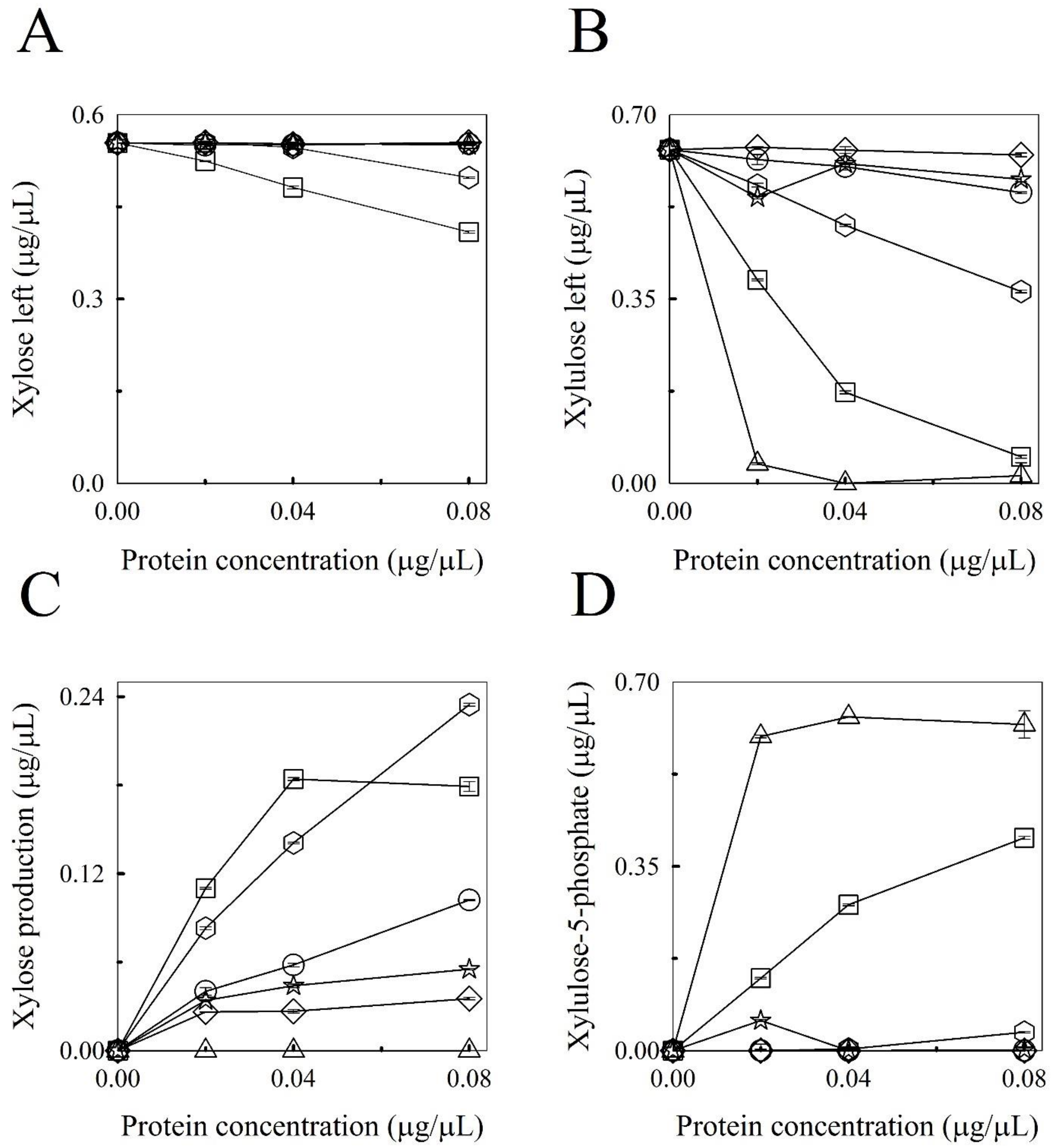

Additional file 2: Figure S10: Xylose isomerase and xylulose kinase activities in wild-type and XI-XK transformants. Experiments were done in triplicate with A) xylose or B) xylulose. Xylose isomerase C) and xylulose kinase D) activities were calculated from the xylulose reactions. The error bars represent standard deviations. Symbols: diamonds, WT; circles, XI 16; triangles, XB; hexagons, XI-XK 1; stars, XI-XK 3; squares, XI-XK 7.

Under these conditions, protein extracts from transformant XB and wild-type T18 had minimal, if any, xylose isomerase activity on xylose (Additional file 2: Figure S10A, Table 2. Very little, if any, xylose isomerase activity was seen with xylose as substrate in protein extracts from XI 16 at 0.08 μg/μL. This activity is lower than what was seen previously for protein extracts from this isolate (Additional file 2: Figure S7A) due to the lower T18 xylose isomerase activity at 30°C and lower protein concentration range tested. However, dose dependency with xylose for protein extracts from XI-XK 1 and XI-XK 7 was observed when incubated with increasing protein amounts (Additional file 2: Figure S10A). The increased activity on xylose for protein extracts from XI-XK 1 and XI-XK 7 is consistent with an increase in xylose isomerase gene copy compared to the parental (Table 2). Minimal, if any, xylose isomerase activity was observed at this protein concentration range in protein extracts from XI-XK 3 consistent with a loss of xylose isomerase genes. Similarly, the gene copy increases and decreases in the protein extracts from XI-XK transformants correlated with activity on xylulose (Additional file 2: Figure S10B, Table 2). Wild-type had minimal activity on the substrate even at 0.08 μg/μL; whereas, protein extracts from transformant XB cell extracts converted close to 94% of the xylulose at 0.02 μg/μL (Additional file 2: Figure S10B). Protein extracts from the parental strain, transformant XI 16, and XI-XK 3 had similar levels of activity on xylulose whereas, protein extracts from both XI-XK 1 and XI-XK 7 had higher activity on xylulose than from the parental strain.

To better dissect the xylose isomerase and xylulose kinase activities in the transformants, the amount of xylulose converted to xylose was used to determine the xylose isomerase activity (Additional file 2: Figure S10C, Table 2). As xylulose-5-phosphate could not be separated under the separation conditions used, xylulose kinase activity in the samples was calculated by deducting the total amount of xylose and xylulose detected in these reactions from the known starting amount of xylulose (Additional file 2: Figure S10D). Based on this analysis, xylose

isomerase and xylulose kinase activities in protein extracts from wild-type T18 were very low. Protein extracts from the parental strain, XI 16, converted 0.10 μg/μL of the xylulose to xylose but had negligible xylulose kinase activity at 0.08 μg/μL total protein. As expected based on gene copy number, the xylose isomerase activity in protein extracts from XI-XK 3 was lower than from the parental strain, XI 16; however, despite having two copies of *aph7-2A-xylB*, there was little xylulose kinase activity observed in this strain. The increased *his-xi* gene copies in XI-XK 1 resulted protein extracts with a 2.3x increase in xylose isomerase activity compared to protein extracts from the parental strain; however, the two *aph7-2A-xylB* copies only resulted in 0.03 μg/μL xylulose converted to xylulose-5-phosphate at 0.08 μg/μL total protein. On the other hand, the gene copy increase of both *ble-2A-his-xi* and *aph7-2A-xylB* in XI-XK 7 correlated well with significant increases in both xylose isomerase and xylulose kinase activities in the protein extracts compared to the parental strain, XI 16 (Table 2).

Additional file 2 References:

1. Hess JM, Tchernajenko V, Vieille C, Zeikus JG, Kelly RM. Thermotoga neapolitana homotetrameric xylose isomerase is expressed as a catalytically active and thermostable dimer in Escherichia coli. Appl. Environ. Microbiol. 1998;64:2357–60.

2. Ben Hlima H, Ayadi D, Aghajari N, Bejar S. Differential properties of native and tagged or untagged recombinant glucose isomerases of Streptomyces sp. SK and possible implication of the glycosylation. J. Mol. Catal. B Enzym. Elsevier B.V.; 2013;94:82–7.
